# Endothelial dysfunction in aging associated with reduced Niban phosphorylation

**DOI:** 10.1007/s11033-026-11504-8

**Published:** 2026-01-29

**Authors:** Brandon Baer, Madeleine Morelli, Colleen Brophy, Julie A. Bastarache, Joyce Cheung-Flynn

**Affiliations:** 1 Department of Medicine, Vanderbilt University Medical Center, Nashville, TN USA; 2 Department of Vascular Surgery, Vanderbilt University Medical Center, Nashville, USA; 3 Department of Cell and Developmental Biology, Vanderbilt University, Nashville, USA; 4 Department of PathologyMicrobiology and Immunology, Vanderbilt University Medical Center, Nashville, USA

**Keywords:** Niban phosphorylation, Age-related vascular dysfunction, Endothelial function, Inflammation-induced vascular injury, Ex vivo, Rats

## Abstract

**Background:**

Cardiovascular diseases are the leading cause of mortality worldwide, with aging and endothelial dysfunction being key contributors to its progression. Age-related vascular dysfunction is characterized by impaired endothelial-dependent relaxation, increased vascular inflammation, and heightened susceptibility to injury, all of which exacerbate cardiovascular risk. The multi-functional protein Niban restores vascular function following injury, with reduced Niban phosphorylation linked to activation of mitogen-activated protein kinase (MAPK) pathways. We hypothesized that reduced Niban phosphorylation and increased inflammatory MAPK signaling would be associated with vascular dysfunction in aging that can be attenuated by NiPp, a cell permeant phosphomimetic peptide of Niban.

**Methods and results:**

Aortas from young (3-months-old, *N* = 8) and aged (20- to 23-month-old *N* = 8) rats were assessed for vascular reactivity as well as protein levels and protein phosphorylation. Aged aortas displayed impaired contractility, endothelial-dependent relaxation, reduced phosphorylated Niban levels, and increased phosphorylation of inflammatory MAPK pathway elements including c-Jun N-terminal kinase, MAP kinase-activated protein kinase 2, phosphorylated cAMP response element-binding protein, and downstream vascular cell adhesion molecule-1. Aged aortas also exhibited greater IL-1β-induced loss of endothelial-dependent relaxation ex vivo, which was attenuated by NiPp treatment.

**Conclusion:**

These results identify reduced Niban phosphorylation and increased MAPK signaling as contributors to age-related endothelial dysfunction and highlight Niban phosphorylation as a possible target for treating vascular aging and associated cardiovascular diseases.

**Supplementary Information:**

The online version contains supplementary material available at 10.1007/s11033-026-11504-8.

## Introduction

Cardiovascular diseases are the leading cause of death worldwide, with aging and endothelial dysfunction being strongly associated with their onset and progression [1–4]. The vascular endothelium, a single layer of endothelial cells lining the inner surface of blood vessels, plays a central role in maintaining vascular health and homeostasis [1, 2, 4]. Age-related endothelial dysfunction is characterized by worse functional viability, impaired endothelial-dependent relaxation, increased inflammation, and heightened vulnerability to injury, all of which contribute to their increased cardiovascular risk [1, 2, 4]. Numerous studies have revealed the contribution of decreased bioavailability of nitric oxide (NO), altered permeability, and oxidative stress [5, 6]. Other endothelial-derived relaxing factors (e.g. prostacyclin) and endothelial-derived hyperpolarization factors (e.g. hydrogen peroxide) may also play a role in impaired endothelial-dilation in a vessel- and disease-dependent manner [7, 8]. Despite substantial evidence linking aging and endothelial dysfunction, the interplay of these pathophysiological factors and the precise molecular mechanisms underpinning this decline remain poorly understood.

Recent studies have highlighted the multifunctional protein Niban as a promising regulator of vascular health and function [9, 10]. Niban activates pathways that regulate apoptosis and endoplasmic reticulum stress responses [11, 12]. The phosphorylated form (p-Niban) protects vascular endothelial cells from inflammation-induced injury [9, 12]. Specifically, in ex vivo models of vascular injury, reduced phosphorylation of Niban was associated with impaired endothelial-dependent relaxation and increased activation of the p38 mitogen-activated protein kinases (MAPK) [9], a key driver of inflammation and cardiovascular diseases [9, 10, 13, 14]. These observations suggest that Niban phosphorylation plays a protective role in the endothelial response to injury and may be critical for preserving vascular function across the lifespan.

To explore the role of Niban phosphorylation in vascular disease, our research group recently developed a novel cell-permeant phosphopeptide mimetic of phosphorylated Niban (NiPp) [9]. NiPp has inhibitory properties against inflammatory p38 MAPK and improves endothelial-dependent relaxation in various ex vivo vascular injury models, including mechanical stretch, acidic saline exposure, and the purinergic P2X7 receptor activation of intact rat aorta [9]. In addition, NiPp improves endothelial relaxation in aged, diseased human saphenous veins collected from patients undergoing coronary artery bypass grafting surgery [9]. Together, these findings suggest that Niban may play a critical role in maintaining vascular homeostasis in pathophysiologic conditions. Moreover, whether Niban activity changes with age and its role in age-related endothelial dysfunction remains unexplored. In this study, we hypothesized that reduced Niban phosphorylation and increased inflammatory MAPK signaling contribute to vascular dysfunction in aging, and that restoring p-Niban levels will attenuate endothelial dysfunction.

## Methods

### Materials

Unless otherwise stated, all chemicals and reagents were obtained from Sigma-Aldrich (St. Louis, MO). The design and synthesis of NiPp followed previously established protocols [9]. Briefly, NiPp (EZ Biolab, Carmel IN) was synthesized using f-moc chemistry and purified via high‐performance chromatography.

### Animals and aorta isolation

Young (3-months-old) and aged (20- to 23-months-old) female Fisher 344 rats were obtained from the National Institute of Aging. Rats were housed in the Vanderbilt University Medical Center animal facility under temperature-controlled conditions with a 12:12 h light/dark cycle and had free access to standard food and water for at least one week prior to experimentation. Following euthanasia via CO₂ exposure, aortic tissues were isolated and immediately used for experiments as described below.

### Measurement of vascular reactivity

Isolated rat aortae were dissected free of perivascular fat, sectioned into rings (1–2 mm thick), and suspended in a muscle bath containing bicarbonate buffer (120 mM sodium chloride, 4.7 mM potassium chloride (KCl), 1.0 mM magnesium sulfate, 1.0 mM monosodium phosphate, 10 mM glucose, 1.5 mM calcium chloride, and 25 mM sodium bicarbonate, pH 7.4) equilibrated with 95% O₂/5% CO₂ at 37 °C. Rings were maintained at a resting tension of 1 g for 1 hour, manually stretched to three times the resting tension, and then returned to resting tension for an additional hour to establish the maximal force-tension relationship, as previously described [9, 10]. Rings were primed with 110 mM KCl (replacing sodium chloride in the bicarbonate buffer) to confirm functional viability and phenylephrine (PE; 10^− 8^ to 10⁻^5^ M) to determine agonist induced contractile responses. Endothelial-dependent and -independent relaxation were assessed by exposing PE-precontracted rings (1–5 × 10^− 7^ M; submaximal contraction at approximately 60%−70% of maximal KCl response) to escalating doses of carbachol (CCH; 10⁻⁸ to 10⁻⁵ M), an acetylcholine analog, and sodium nitroprusside (SNP; 10^− 10^ to 10^− 6^ M), respectively. Force measurements were recorded using a Radnoti force transducer (model 159901 A; Radnoti LLC) interfaced with a PowerLab data acquisition system and Chart software (AD Instruments Inc., Colorado Springs, CO). Contractile responses were defined by stress, calculated by normalizing force generated by tissues to the tissue’s length and weight (×10^5^ N/m^2^ = force (g) × 0.0987/area, where area = wet weight (mg)/at maximal length (mm)]/1.055) [9], and percent relaxation was expressed as the change in stress relative to the maximal PE-induced contraction (set as 100%). To determine responses to inflammatory insult, additional rings were incubated in buffer or the inflammatory mediator, interleukin-1β (IL-1β; 50 ng/ml; LifeSpan Bioscience, Seattle, WA), in the absence or presence of NiPp (100 µM) for 2 hours after KCl contraction, and responses to CCH and SNP were measured in PE pre-contracted tissues.

### Western blot analysis

Aortic tissues were snap-frozen in liquid nitrogen and homogenized prior to protein extraction using modified RIPA buffer (Millipore, Burlington, MA). Protein samples were separated via SDS-PAGE and transferred to nitrocellulose membranes. Membranes were blocked at room temperature for 1 hour using Intercept Blocking Buffer (Li-Cor, Lincoln, NE), followed by overnight incubation at 4 °C with primary antibodies specific for phosphorylated and total proteins from Protein Tech (Rosemont, IL): Niban (ProteinTech; Rabbit; 21333-1AP), ThermoFisher (Waltham, MA): heat shock protein 27 (HSP27; Rabbit; PA1-016), Santa Cruz Biotechnology (Dallas, TX): p-HSP27 (Mouse; 13132), Cell Signaling (Danvers, MA): p38 MAPK (mouse; 9228), p-p38 MAPK (Rabbit; 9211), extracellular-signal-regulated kinase (ERK; Rabbit; 9101), p-ERK (Rabbit; 9102), c-Jun N-terminal kinases (JNK; Rabbit; 9252), p-JNK (Mouse; 9255), MAPK-activated protein kinase 2 (MK2; Rabbit; 12155), p-MK2 (Rabbit; 3007) cAMP response element-binding protein (CREB; Mouse; 9104), and p-CREB (Rabbit; 9198), and R&D Signaling (Minneapolis, MN): Vascular Cell Adhesion Molecule-1 (VCAM-1; Mouse; AF643). Polyclonal antibodies against phosphorylated Niban were generated by the Vanderbilt Antibody and Research Core immunizing rabbits with a synthetic peptide containing the sequence surrounding the phosphorylated serine 602 of Niban conjugated to KLH, followed by purification of the resulting antibodies from rabbit serum. After primary antibody incubation, membranes were treated with IRDye-labeled secondary antibodies (Li-Cor Biosciences, Lincoln, NE) and visualized using the Odyssey Infrared Imaging System. Protein levels were normalized to tubulin (Mouse, Sigma; T9026) or GAPDH (Mouse; Millipore; MAB374), while phosphorylation levels were calculated as the ratio of phosphorylated protein to total protein.

### Statistical analysis

Vascular reactivity data were presented as an average of at least 2 rings. Data were analyzed using GraphPad Prism 10 and presented as individual values or mean *±* standard deviation. Comparisons between two groups were performed using the Mann-Whitney test, while repeated two-way ANOVA with Sidak’s multiple comparisons test was used for dose-dependent analyses between groups. For comparisons involving two or more groups across multiple doses of dependent (matched) samples from the same animal, repeated two-way ANOVA followed by Tukey’s multiple comparisons test was employed. Statistical significance was defined as a *p* < 0.05.

## Results

### Aged rat aortas have impaired contractility and endothelial-dependent relaxation

**Fig. 1:**
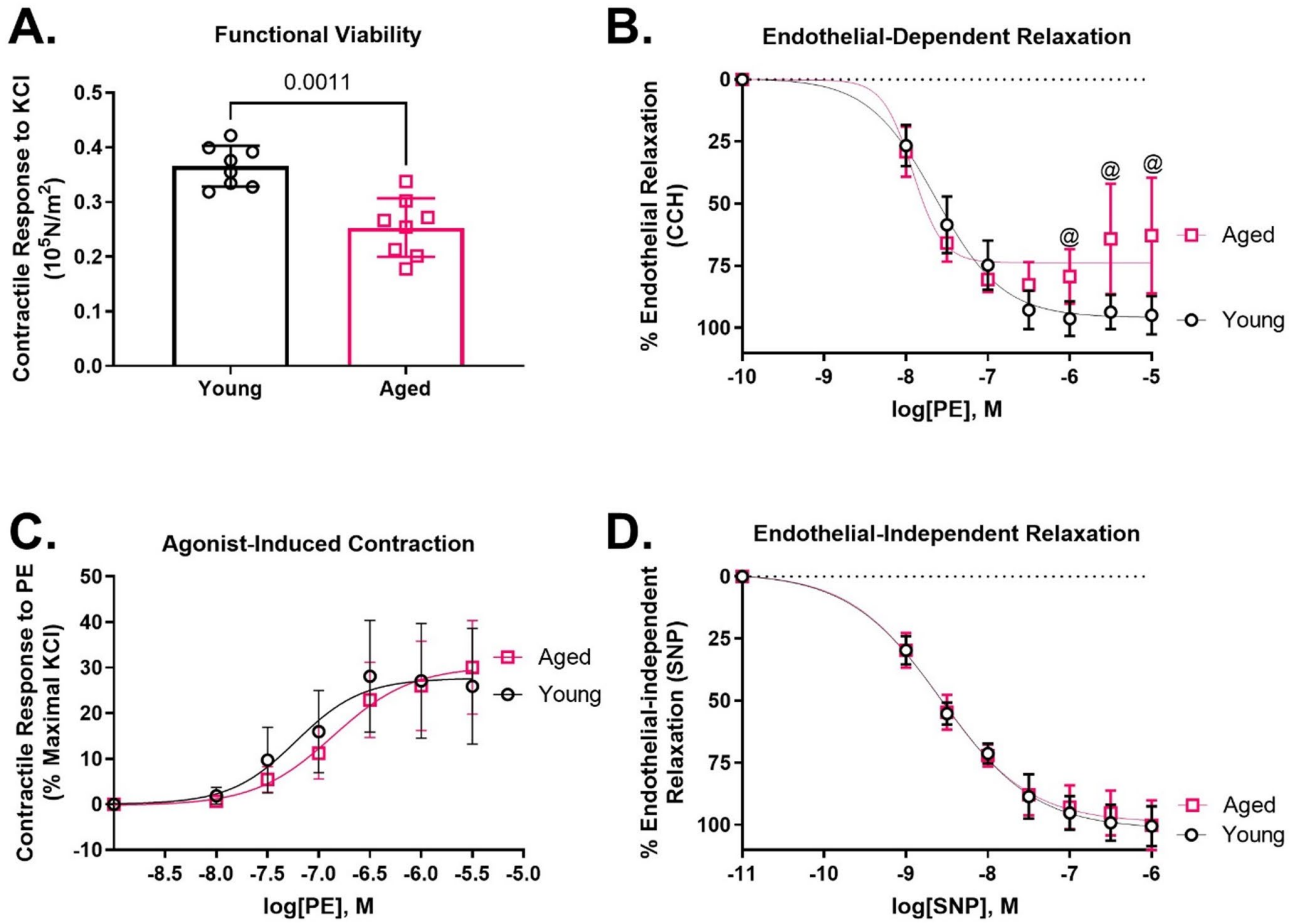
Vascular reactivity of aortas isolated from aged (20- to 23-months-old) and young (3-month-old) rats. Aged rat aortas had impaired KCl-induced contraction (**A**) and percent endothelial-dependent relaxation (**B**) compared to young rat aortas. No differences were observed for contraction (% Maximal KCl) induced by phenylephrine (PE; **C**) or percent endothelial-independent relaxation in response to sodium nitroprusside (SNP; **D**). Data were presented as individual data points each representing an individual animal or mean +/- SD (**A**-**D**). Lines represent non-linear regression line of best fit (**B**-**D**). ^@^*p* < 0.05 young vs. aged. *N* = 7–8. [Statistical analysis: Mann-Whitney test (**A**); Two-way ANOVA + Sidak’s multiple comparisons test (**B**-**D**)]. CCH = carbachol, KCl = potassium chloride, SD = standard deviation

At baseline, aged rat aortas had decreased contractile response to KCl (0.366 ± 0.037 vs. 0.253 ± 0.054 × 10^5^ N/m^2^; Fig. 1A) and impaired endothelial-dependent relaxation in response to CCH, compared to young rat aortas (maximal relaxation 94.84 ± 7.58 vs. 62.76 ± 21.22%; Fig. 1B), indicating worse functional viability and endothelial function. No differences were observed between young and aged rat aortas for contraction to PE (maximal contraction 25.90 ± 12.71 vs. 30.8 ± 10.26%; Fig. 1C) or endothelial-independent relaxation in response to SNP (maximal relaxation 100.394 ± 7.86 vs. 100.06 ± 9.92%; Fig. 1D).

### Aged rat aortas have reduced Niban phosphorylation and increased phosphorylation of components in the MAPK inflammatory pathways

**Fig. 2:**
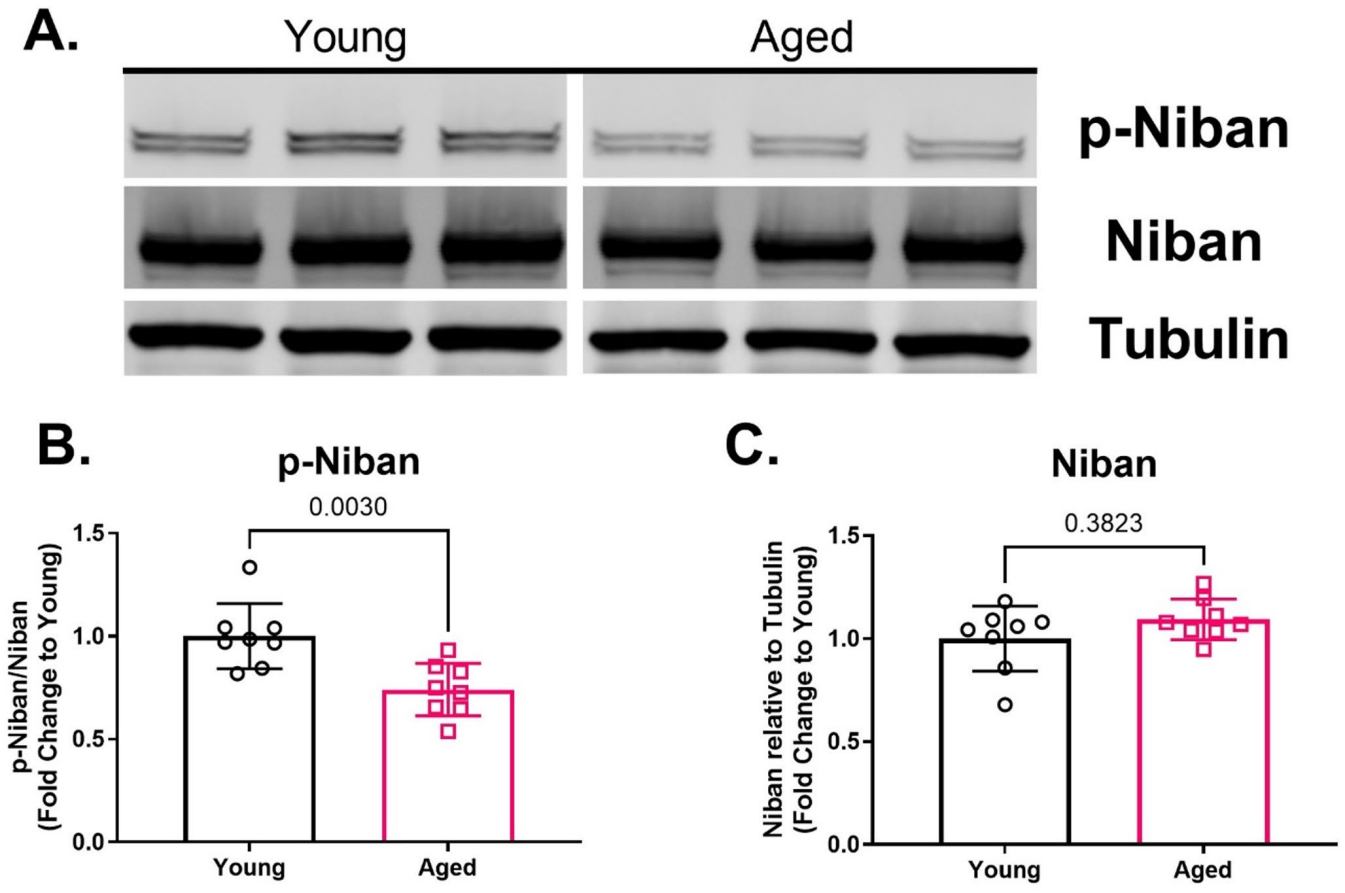
Protein levels for phosphorylated Niban and total Niban in aortas isolated from young (3-months-old) and aged (20- to 23-months-old) rats. Representative Western blots for p-Niban, Niban, and tubulin (**A**). Aortas from aged rats showed reduced Niban phosphoryaltion (**B**) without altering protein levels of total Niban (**C**) compared to young rat aortas. Protein levels were presented as fold-change relative to young rat aortas, while data were presented as individual data points each representing an individual animal (**B**-**C**). Bars represent mean and error bars represent standard deviation. *N* = 8. [Statistical analysis: Mann-Whitney test (**B**-**C**)]. p-Niban = phosphorylated Niban

**Fig. 3:**
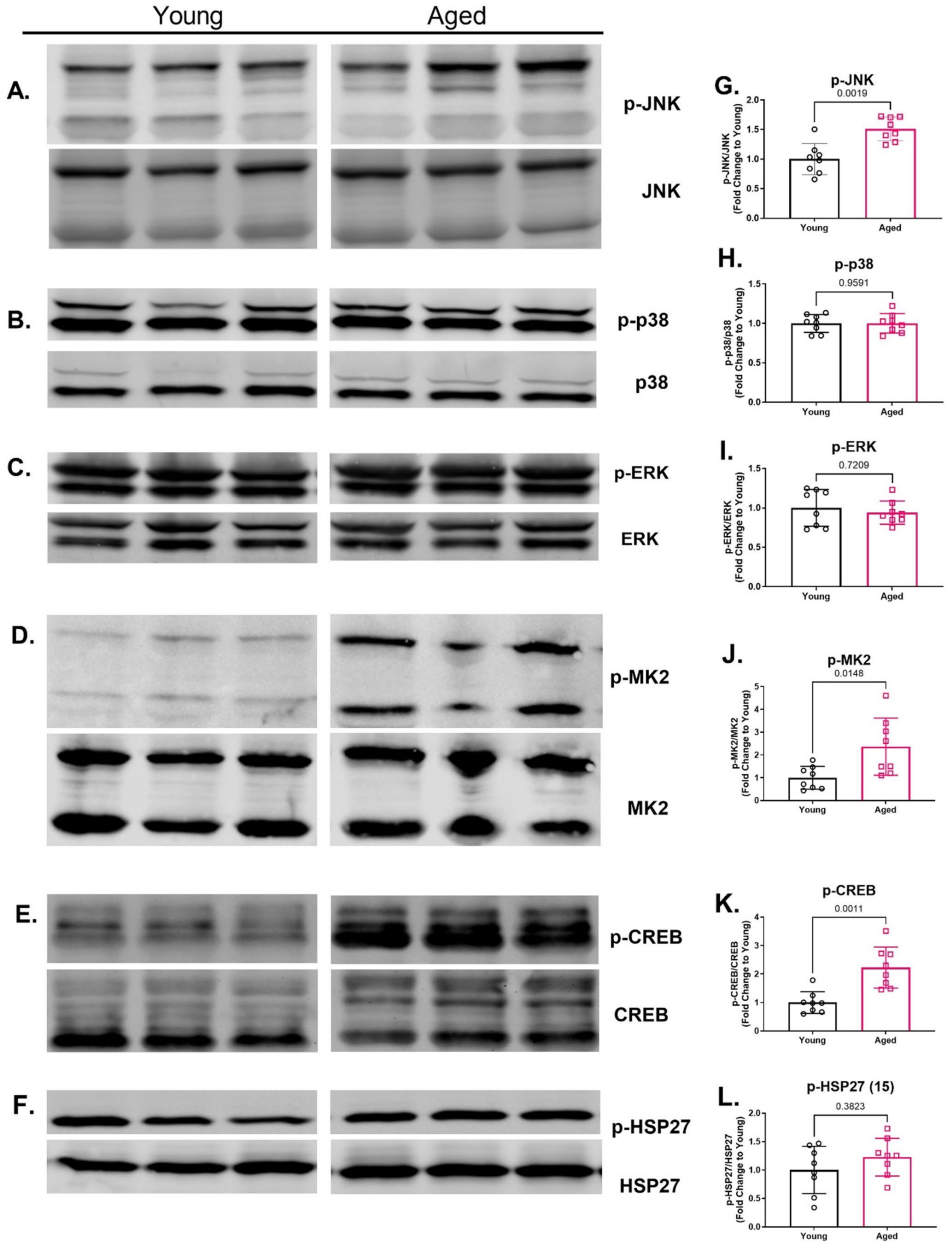
Protein levels for stress-activated kinases and transcription factors in the MAPK pathways for aortas isolated from aged (20- to 23-months-old) and young (3-months-old) rats. Representative Western blots for p-JNK, JNK (**A**), p-p38, p38 (**B**), p-ERK, ERK (**C**), p-MK2, MK2 (**D**), p-CREB, CREB (**E**), p-HSP27, and HSP27 (**F**). Aged rat aortas exhibited higher protein levels of phosphoryated JNK (**G**), MK2 (**J**) and CREB (**K**) compared to young rat aortas. Compared to young rat aortas, aged rat aortas also had a numerical higher protein levels of phosphorylated HSP27 (serine 15) (**L**). No differences in phosphorylation levels were observed between young and age rat aortas for p-38 (**H**) or ERK (**I**). Data were presented as individual data points each representing an individual animal (**G**-**L**). Bars represent mean and error bars represent standard deviation. *N* = 8. [Statistical analysis: Mann-Whitney test (**G**-**L**)]. MAPK = mitogen-activated protein kinase, JNK = c-Jun N-terminal kinases, ERK = extracellular-signal-regulated kinase, MK-2 = MAPK-activated protein kinase 2, CREB = cAMP response element-binding protein, HSP27 = heat shock protein 27, p-ERK= phosphorylated ERK, p-JNK = phosphorylated JNK, p-MK2 = phosphorylated MK2, p-CREB = phosphorylated cAMP response element-binding protein, p-HSP27 = phosphorylated heat shock protein 27

**Fig. 4:**
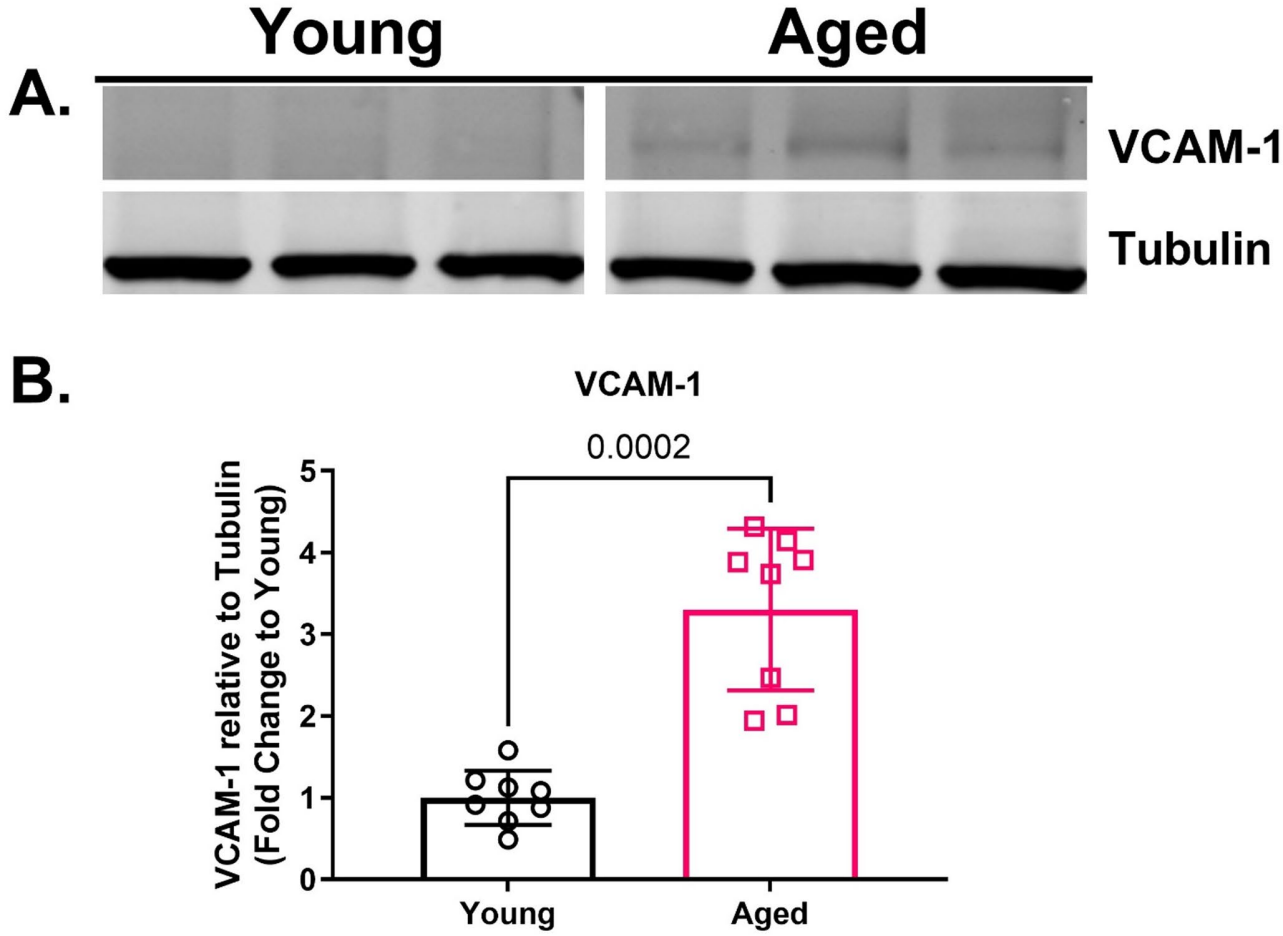
Protein levels for vascular cell adhesion molecule 1 (VCAM-1) in aortas isolated from aged (20- to 23-months-old) and young (3-months-old) rats. Representative Western blots for VCAM-1 and tubulin (**A**). Aged rat aortas had higher protein levels of VCAM-1 (**B**) compared to young rat aortas. Data were presented as individual data points each representing an individual animal (**B**). Bars represent mean and error bars represent standard deviation. *N* = 8. [Statistical analysis: Mann-Whitney test (**B**)]

Western blot analysis revealed that aged rat aortas had decreased baseline levels compared to young rat aortas for phosphorylated Niban without any change in total Niban protein (Fig. 2B-C). Aged rat aortas also exhibited increased phosphorylation of JNK MAPK, but no differences in phosphorylated p38 MAPK or ERK MAPK compared to young rat aortas (Fig. 3G-I). Phosphorylation of downstream effectors in the p38 MAPK inflammatory signaling, including phosphorylated-MK2 and phosphorylated-CREB were significantly elevated in aged aortae, while phosphorylated HSP27 (serine 15) was numerically higher compared to young aortae (Fig. 3J-L). Moreover, aged aortas displayed elevated protein levels of VCAM-1 compared to young aortas (Fig. 4B). Aged aortas also displayed lower protein levels for total p-38 MAPK and higher protein levels for total HSP27, with no differences across total ERK, JNK, MK-2 or CREB (Supplemental Fig. 1G-L).

### Aged rat aortas show worse inflammation-induced endothelial dysfunction, which was attenuated by NiPp treatment

**Fig. 5:**
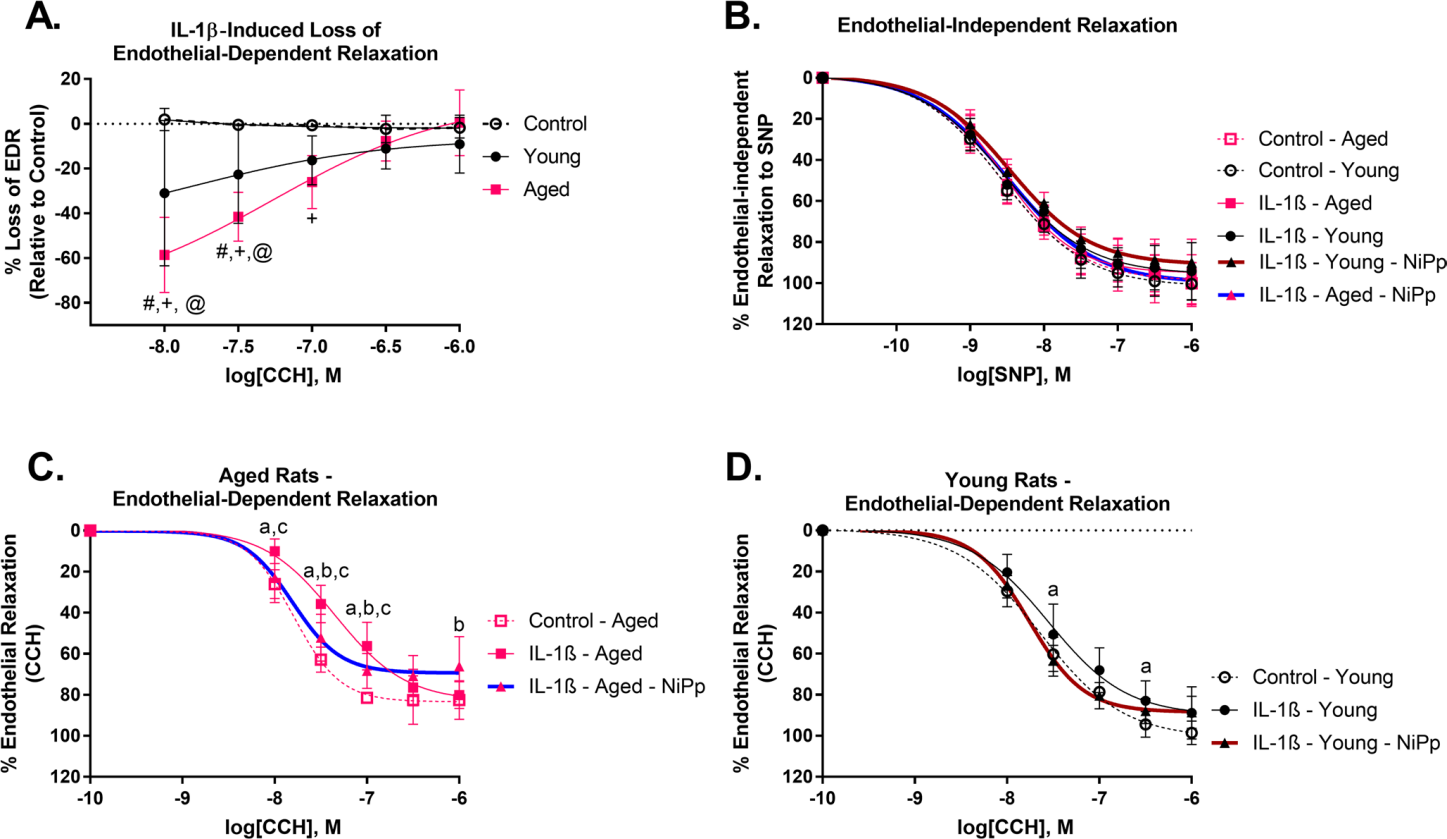
Effect of an inflammatory insult and NiPp treatment on endothelial dependent relaxation and endothelial-independent relaxation in aortas isolated from young (3-month-old) and aged (20- to 23-months-old) rats. Aged rat aortas displayed a greater IL-1β-induced loss of endothelial-dependent relaxation (%) compared to young rat aortas (**A**). Exposure to IL-1β or NiPp treatment did not affect endothelial-independent relaxation regardless of age (**B**). NiPp treatment attenuated IL-1β-induced loss of endothelial-dependent relaxation in aortas isolated from aged (**C**), but not young rats (**D**). Data were presented as mean +/- SD, with non-linear regression line of best fit (**A-D**). *N* = 8. [Statistical analysis: Two-way ANOVA + Sidak’s multiple comparison tests (**A-B**); Repeated two-way ANOVA + Tukey’s multiple comparison tests (**C-D**)]. #p < 0.05 young vs. control, +p < 0.05 aged vs. control, @p < 0.05 aged vs. young, ^a^p < 0.05 IL-1β vs. control, ^b^p < 0.05 IL-1β-NiPp vs. control, ^c^p < 0.05 IL-1β vs. IL-1β-NiPp. IL-1β = interleukin-1β, EDR= endothelial-dependent relaxation, CCH = carbachol, SNP = sodium nitroprusside, SD = standard deviation

Since the phosphorylation of MAPK inflamamtory pathways were elevated in aged aortas, we next determined if responses to inflammatory insults were also altered. Exposure to IL-1β impaired endothelial-dependent relaxation in both young and aged aortae (Fig. 5A, C-D). Endothelial-independent relaxation was simlar between young and aged aortae regardless of IL-1β or NiPp treatment (Fig. 5B). Compared to young rat aortas, aged rat aortas had more pronounced impariments in endothelial-dependent relaxation in response to IL-1β (Fig. 5A), which was partially attenuated with NiPp treatment (Fig. 5C). The protective effects of NiPp were not detected in young aortae exposed to IL-1β (Fig. 5D).

## Discussion

Vascular dysfunction and chronic low-grade inflammation are hallmarks of aging and critical contributors to the development of cardiovascular pathologies in the elderly population [15]. In this study, we investigated the impact of aging on aortic endothelial dysfunction, focusing on the role of Niban phosphorylation and the MAPK inflammatory signaling pathway in young (3-month-old) and aged (20- to 23-month-old) rats. Our findings provide novel insights into the molecular mechanisms underlying vascular aging and suggest potential therapeutic strategies for mitigating age-associated vascular dysfunction.

The effects of aging on vascular contractile responses to KCl and PE have been reported with considerable variability across studies. In the current study, aging significantly impaired contractile response of smooth muscle to KCl, while PE-induced contraction remained unchanged in aged rat aortas. These findings align with previous observations in F344 rat aorta [16], as well as basilar arteries from rats and humans [17, 18], suggesting that KCl-induced contractility may decline with age in certain vascular beds. Furthermore, as cellular viability correlates with functional viability [19], these results may infer an increase in smooth muscle cell death in the aged aortas. However, contrasting evidence exists, such as studies showing no significant difference in KCl-induced contractility between young and aged aortic strips from Wister rats [20], rabbits [21], and in accelerated senescence mouse models [22, 23]. Similarly, some studies reported diminished adrenergic receptor induced contraction with age, while responses to KCl remained unchanged [24], underscoring variability across vascular beds and species. These conflicting results highlight the complexity of age-related changes in vascular contractile function, which may depend on factors such as potassium channel activity, calcium homeostasis, and intracellular signaling pathways [25–28]. This complexity emphasizes the need for further research to elucidate the multifaceted effects of aging on vascular physiology.

The vascular endothelium plays a critical role in maintaining vascular homeostasis, and age-related endothelial dysfunction has been consistently reported [1, 2, 4]. In our study, aged rat aortas exhibited a reduced maximal relaxation compared to young rat aortas, while endothelial-independent relaxation remained unaffected. Moreover, endothelial dysfunction was associated with increased MAPK inflammatory signaling and endothelial activation in the absence of injury or infection. Specifically, we observed elevated levels of phosphorylated JNK MAPK in aged rat aortas compared to young rat aortas, while the levels of phosphorylated ERK and p38 MAPKs remained unchanged. These data align with previous findings in aged rat aortas [29] and the established role of JNK in “death” signaling, where it responds to both extrinsic and intrinsic stimuli, including cytokines and oxidative stress, both of which are elevated with aging and contribute to cellular senescence [30]. Tissue specific JNK activity also increases with age, promoting age-related pathologies such as macrovascular and microvascular diseases [30–32]. The observed increase in phosphorylated JNK may contribute to vascular dysfunction in aging by enhancing apoptotic signaling, potentially explaining the diminished reactivity of aged aortas to KCl in our study. The p38 MAPK pathway, which regulates the production of inflammatory cytokines such as IL-1β, through post-transcriptional mechanisms involving MK2 and CREB [33–36], did not show age-related differences in basal phosphorylation levels, despite increases in phosphorylated MK2 and CREB in aged rat aorta. VCAM-1, a key component of the inflammatory cascade, facilitates the selective adhesion of monocytes and lymphocytes, and is known to be upregulated by both JNK and MK2 pathways [36, 37]. This suggests that the JNK and p38 MAPK pathways may interact to amplify inflammation in aged vascular tissue. Additionally, the interplay between these MAPKs and IL-1β can create a feed-forward loop that perpetuates chronic low-grade inflammation, further implicating these pathways in the aged-related vulnerability of the vascular endothelium [38]. Taken together, these findings suggest that this sustained inflammatory state resulting from dysregulated MAPK inflammatory signaling may render the aged vascular endothelium more susceptible to pro-inflammatory insults, accelerating the progression and severity of vascular pathologies in the elderly.

Precise regulation of kinase cascades is crucial to cellular homeostasis, and disruptions in these regulatory mechanisms have been implicated in aging. For instance, age-associated changes in protein phosphatases, which serve as the “off” signal for kinase activation, have been proposed to explain the decline in the responsiveness of AMP-activated protein kinase signaling with age [39]. Since, the phosphomimetic of Niban, NiPp, has inhibitory effects on p38 MAPK in rat aorta and human endothelial cells [9], Niban phosphorylation may serve as an endogenous “off” signal for MAPK signaling. Notably, reduced Niban phosphorylation has been linked to acute vascular injury, which is associated with the activation of p38 MAPK, but not ERK [9]. In the current study, age rat aortas exhibited reduced basal Niban phosphorylation in the absence of age-related changes in phosphorylated p38 MAPK compared to young rat aortas, suggesting that aging may disrupt the regulatory role of Niban under chronic inflammatory conditions. This discrepancy between acute and chronic states suggests that MAPK activity is age- and context-dependent, varying under different stress conditions, and it highlights the complexity of Niban regulation. This intricate interplay between Niban and MAPK signaling, along with its implications for vascular health and aging, warrant further investigation.

The use of intact aged aorta in the muscle bath provided a controlled system to evaluate baseline endothelial dysfunction and its response to inflammatory insults, free from systemic influences, such as hormonal fluctuations, immune cell interactions, and circulating inflammatory mediators [4, 40–42] This ex vivo approach also facilitated the identification of intrinsic aorta-specific defects associated with vascular aging and manipulation of endogenous signaling through cell permeant phosphomimetic. Direct exposure of the aortas to IL-1β revealed that aged aortas exhibited greater severity of endothelial dysfunction than their young counterpart, likely due to age-related priming of the MAPK inflammatory signaling network and reduced Niban activity. Notably, treatment with NiPp attenuated the IL-1β-induced endothelial dysfunction in aged aortas, suggesting that restoring intracellular level of activated Niban has salutary effects on vascular function and that reduced Niban phosphorylation plays a role in the exacerbation of age-related endothelial dysfunction induced by an inflammatory insult. NiPp treatment did not improve endothelial function in young aortas exposed to IL-1β, likely due to the minor loss of endothelial function in the absence of aging-related stress. Multiple models of vascular injury have demonstrated that NiPp treatment reduces p38 MAPK phosphorylation [9], suggesting that the partial restoration of IL-1β-induced endothelial dysfunction in aged rat aortas by NiPp is, at least in part, attributable to suppression of p38 MAPK activity. While elevated JNK phosphorylation was detected in the aged aortas and IL-1β can induce transient JNK activation [34, 36], NiPp does not inhibit JNK or ERK in kinase screening and rat aortas treated with a p38 MAPK activator ex vivo [9].

**Fig. 6:**
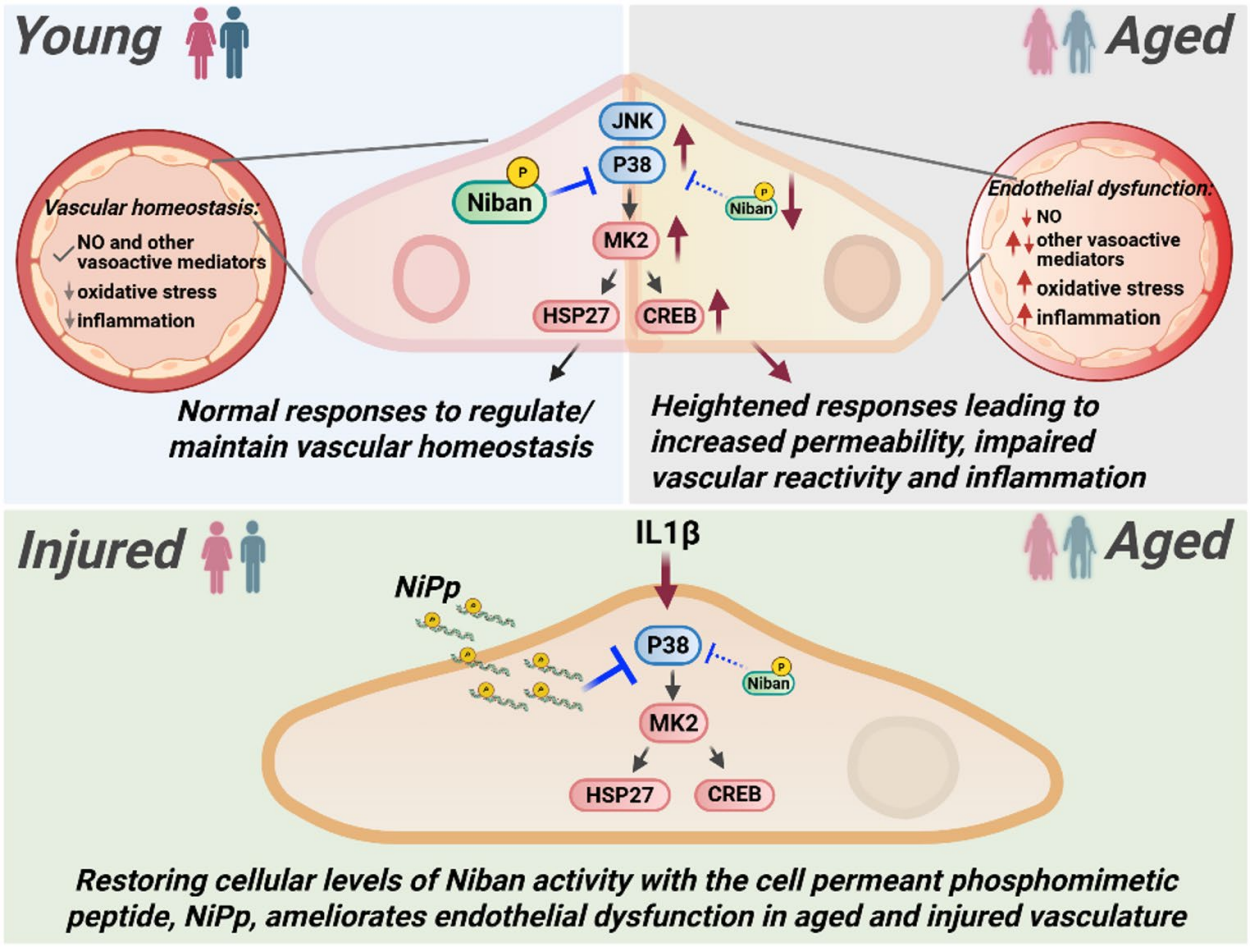
Proposed role of Niban in vascular health, aging, and injury. In younger vasculature (top left panel), vascular homeostasis is maintained through normal levels of nitric oxide (NO) and other vasoactive mediators, as well as limited oxidative stress and inflammation. Niban activity supports normal p38 MAPK inflammatory pathway signaling in the enodthelial cells. In aged vessles (top righ panel), dimnished Niban activity results in heightened MAPK signaling, contributing to endothelial dysfucntion characterized by altered levels of NO and other vasoative mediators, increased permeabiltiy, oxidative stress, inflammation, and impaired vascular reactivity. Injury by acute inflammatory stimuli (e.g. IL-1β), further exacerbate endothelial dysfunction (bottom panel). Treatment with NiPp restores intracellular Niban activity, attenuating p38 MAPK signaling and ameliorating endothelial dysfucntion in aged and injured vasculature. NO = nitric oxide, MAPK = mitogen-activated protein kinase, IL-1β = interleukin-1β

Collectively, our findings support the hypothesis that reductions in phosphorylated Niban are associated with exacerbated endothelial injury and inflammation in aging, disrupting vascular homeostasis (Fig. 6). NiPp, by mimicking phosphorylated Niban, may also offer a targeted therapeutic strategy to restore regulation of the inflammatory MAPK pathway and mitigate stress-induced endothelial dysfunction [14, 43–45]. Unlike broad anti-inflammatory agents or MAPK inhibitors, NiPp functions to specifically mimic endogenous Niban activity, providing a more focused approach to reducing inflammation and enhancing endothelial resilience. Additionally, the protective effects of NiPp against inflammatory stress in aged vessels suggest its potential utility in conditions involving acute inflammation, such as sepsis [46], as well as chronic inflammatory states like advanced aging [6, 15], where heightened baseline and exaggerated inflammatory responses increase susceptibility to insult.

The progressive pathogenesis behind aged-related endothelial dysfunction involves multiple cellular mechanisms which are often interrelated to shape vascular aging. A number of molecular pathways involved in these pathologic changes have been previously identified and form the bases of intervention strategies to mitigate vascular aging, including the NAPDH oxidases, NF-κB, JAK/STAT, AMPK, mTOR, Klotho, sirtuins, p53/P21 signaling that influences NO balance, oxidative stress, senescence, and mitochondrial quality control/autophagy [47–49]. Here in this study, we identified an additional intrinsic defect associated with vascular aging [2–4], reduced Niban phosphorylation and exaggerated responses to inflammatory stress. Moreover, Niban has been implicated in some of these processes involving apoptosis and autophagy [50]. Thus, by targeting these defects, NiPp is both a valuable research tool to further understanding of the cellular and molecular mechanisms of vascular aging, and a promising therapeutic candidate to reduce higher morbidity and mortality associated with cardiovascular dysfunction in the elderly [1–4].

While this study provides valuable insights into the role of Niban in vascular aging, several limitations must be acknowledged. First, only female rats were used in this study. Female rats have been exclusively used by others to study vascular function and thus provide relevance to examine the role of Niban in vascular homeostasis [51–53]. While this allowed for a focused investigation of age-related changes, it precluded the investigation of sex-based differences in molecular alterations and vascular responses. Second, this study focused on IL-1β as an inflammatory stimulus to induce aortic dysfunction. Although IL-1β is widely used and relevant for modeling inflammation-driven vascular changes [10], it represents only one of many age-related physiological stressors, leaving unanswered questions about how other stimuli, such as oxidative stress, might interact with Niban signaling. Third, while reduced Niban phosphorylation has emerged as a common feature of endothelial dysfunction induced by different injuries ex vivo [9, 10], the precise mechanisms through which Niban regulates age-related endothelial dysfunction and MAPK signaling remain unclear and require further investigation. Additionally, the ex vivo approach, while highly controlled and informative, does not fully capture the complexity of the in vivo environment. In vivo studies will therefore be essential to validate our findings, determine the broader implications of Niban phosphorylation, and assess the therapeutic potential of NiPp. Overall, future studies will aim to address these limitations by incorporating aged models of both sexes, validating the efficacy of NiPp in vivo, and exploring other hallmark features of vascular aging [5–8], such as oxidative stress, mitochondrial dysfunction, autophagy, and senescence pathways [3]. These efforts will provide a more comprehensive understanding of the role of Niban in vascular aging.

In conclusion, this study identifies reduced phosphorylation of Niban as a molecular feature associated with age-related endothelial dysfunction and highlights the therapeutic potential of restoring Niban phosphorylation to improve endothelial response to stressors in aged aortas. Additionally, by targeting dysregulated signaling pathways, such as inflammatory MAPK signaling, NiPp offers a focused therapeutic strategy for mitigating vascular dysfunction in aging populations. These findings advance our understanding of molecular mechanisms underlying vascular aging and pave the way for future research aimed at improving vascular health in aging individuals.

## Supplementary Information

Below is the link to the electronic supplementary material.

Supplementary material 1 (DOCX 352.2 kb)

Supplementary material 2 (PDF 2107.3 kb)

## Data Availability

All data are provided within the manuscript or supplementary information files.

## Abbreviations

p-Niban: phosphorylated Niban
MAPK: mitogen-activated protein kinases
NiPp: cell-permeant phosphopeptide mimetic of phosphorylated Niban
KCl: potassium chloride
PE: phenylephrine
CCH: carbachol
SNP: sodium nitroprusside
HSP27: heat shock protein 27
ERK: extracellular-signal-regulated kinase
JNK: c-Jun N-terminal kinases
MK2: MAPK-activated protein kinase 2
CREB: cAMP response element-binding protein
VCAM-1: Vascular Cell Adhesion Molecule-1

## References

[1] Donato AJ, Machin DR, Lesniewski LA (2018) Mechanisms of dysfunction in the aging vasculature and role in age-related disease. Circ Res 123:825–848. 10.1161/CIRCRESAHA.118.312563/ASSET/AC23828A-DC27-4DAD-AC4B-0EB4467243CD/ASSETS/IMAGES/LARGE/825FIG06.JPG 30355078 PMC6207260

[2] Moturi S, Ghosh-Choudhary SK, Finkel T (2022) Cardiovascular disease and the biology of aging. J Mol Cell Cardiol 167:109–117. 10.1016/j.yjmcc.2022.04.005 35421400 10.1016/j.yjmcc.2022.04.005

[3] Almeida AD, Ribeiro TP, Medeiros ID (2017) Aging: molecular pathways and implications on the cardiovascular system. Oxid Med Cell Longev 2017:7941563. 10.1155/2017/7941563 28874954 10.1155/2017/7941563 PMC5569936

[4] Nilsson Wadström B, Persson M, Engström G, Nilsson PM (2022) Aortic Stiffness, Inflammation, and incidence of cardiovascular events in elderly participants from the general population. Angiology 73:51–59. 10.1177/00033197211017406/SUPPL_FILE/SJ-DOCX-1-ANG-10.1177_00033197211017406.DOCX 34013787 10.1177/00033197211017406

[5] Jia G, Aroor AR, Jia C, Sowers JR (2019) Endothelial cell senescence in aging-related vascular dysfunction. Biochim Biophys Acta Mol Basis Dis 1865:1802–1809. 10.1016/j.bbadis.2018.08.008 31109450 10.1016/j.bbadis.2018.08.008

[6] Bermejo-Martin JF, Martín-Fernandez M, López-Mestanza C, Duque P, Almansa R (2018) Shared features of endothelial dysfunction between sepsis and its preceding risk factors (Aging and chronic Disease). J Clin Med 7:400. 10.3390/JCM7110400 30380785 10.3390/jcm7110400 PMC6262336

[7] Drachuk K, Nishijima Y, Parthasarathy A, Xie Y, Nagavally S, Dawson A, Gutterman DD, Zhang DX (2025) The role of NO, H_2_O_2_, and non-NO/H_2_O_2_ mechanisms in acetylcholine (ACh)-induced dilation of human arterioles in the absence and presence of coronary artery disease. Basic Res Cardiol. 10.1007/S00395-025-01143-8 41176511 10.1007/s00395-025-01143-8 PMC13046465

[8] Çelik MC, Kalçık M, Birgün A, Yetim M, Bekar L, Karavelioğlu Y (2025) Endothelial dysfunction and vascular stiffness: molecular drivers of cardiovascular aging. Open Explor 2019 3(3):101279. 10.37349/EC.2025.101279

[9] Yim TW, Perling D, Polcz M, Komalavilas P, Brophy C, Cheung-Flynn J (2020) A cell permeant phosphopeptide mimetic of Niban inhibits p38 MAPK and restores endothelial function after injury. FASEB J 34:9180. 10.1096/FJ.201902745R 32396246 10.1096/fj.201902745R PMC7383822

[10] Luo W, Feldman D, McCallister R, Brophy C, Cheung-Flynn J (2017) P2X7R antagonism after subfailure overstretch injury of blood vessels reverses vasomotor dysfunction and prevents apoptosis. Purinergic Signal 13:579. 10.1007/S11302-017-9585-0 28905300 10.1007/s11302-017-9585-0 PMC5714848

[11] Tang S, Wang J, Liu J, Huang Y, Zhou Y, Yang S, Zhang W, Yang M, Zhang H (2019) Niban protein regulates apoptosis in HK-2 cells via caspase-dependent pathway. Ren Fail 41:455–466. 10.1080/0886022X.2019.1619582 31163002 10.1080/0886022X.2019.1619582 PMC6566711

[12] Sun GD, Kobayashi T, Abe M, Tada N, Adachi H, Shiota A, Totsuka Y, Hino O (2007) The endoplasmic reticulum stress-inducible protein Niban regulates eIF2α and S6K1/4E-BP1 phosphorylation. Biochem Biophys Res Commun 360:181–187. 10.1016/j.bbrc.2007.06.021 17588536 10.1016/j.bbrc.2007.06.021

[13] Corre I, Paris F, Huot J (2017) The p38 pathway, a major pleiotropic cascade that transduces stress and metastatic signals in endothelial cells. Oncotarget 8:55684. 10.18632/ONCOTARGET.18264 28903453 10.18632/oncotarget.18264 PMC5589692

[14] Ng GYQ, Loh Z-L, Fann DY, Mallilankaraman K, Arumugam TV, Hande MP (2024) Role of Mitogen-Activated Protein (MAP) Kinase Pathways in Metabolic Diseases. Genome Integr 15:e20230003. 10.14293/GENINT.14.1.004 38770527 10.14293/genint.14.1.004 PMC11102075

[15] Liberale L, Montecucco F, Tardif JC, Libby P, Camici GG (2020) Inflamm-ageing: the role of inflammation in age-dependent cardiovascular disease. Eur Heart J 41:2974. 10.1093/EURHEARTJ/EHZ961 32006431 10.1093/eurheartj/ehz961 PMC7453832

[16] Shipley RD, Muller-Delp JM (2005) Aging decreases vasoconstrictor responses of coronary resistance arterioles through endothelium-dependent mechanisms. Cardiovasc Res 66:374–383. 10.1016/J.CARDIORES.2004.11.005 15820206 10.1016/j.cardiores.2004.11.005

[17] Tümer N, Toklu HZ, Muller-Delp JM, Oktay Ş, Ghosh P, Strang K, Delp MD, Scarpace PJ (2014) The effects of aging on the functional and structural properties of the rat basilar artery. Physiol Rep 2:e12031. 10.14814/PHY2.12031 24907295 10.14814/phy2.12031 PMC4208653

[18] Hatake K, Wakabayashi I, Kakishita E, Hishida S (1992) Effect of aging on contractile response to KCl, norepinephrine and 5-hydroxytryptamine in isolated human basilar artery. Gen Pharmacol 23:417–420. 10.1016/0306-3623(92)90104-R 1511850 10.1016/0306-3623(92)90104-r

[19] Hocking KM, Brophy C, Rizvi SZ, Komalavilas P, Eagle S, Leacche M, Balaguer JM, Cheung-Flynn J (2010) Detrimental effects of mechanical stretch on smooth muscle function in saphenous veins. Journal of vascular surgery : official publication, the Society for Vascular Surgery [and] International Society for Cardiovascular Surgery, North American Chap 53:454. 10.1016/J.JVS.2010.09.010 10.1016/j.jvs.2010.09.010 PMC3053010 21146345

[20] Heymes C, Habib A, Yang D, Mathieu E, Marotte F, Samuel JL, Boulanger CM (2000) Cyclo-oxygenase-1 and – 2 contribution to endothelial dysfunction in ageing. Br J Pharmacol 131:804. 10.1038/SJ.BJP.0703632 11030731 10.1038/sj.bjp.0703632 PMC1572389

[21] Cupitra NI, Calderón JC, Narvaez-Sanchez R (2020) Influence of ageing on vascular reactivity and receptor expression in rabbit aorta: a complement to elastocalcinosis and smooth muscle mechanisms. Clin Interv Aging 15:537. 10.2147/CIA.S236173 32368020 10.2147/CIA.S236173 PMC7182455

[22] Novella S, Dantas AP, Segarra G, Novensa L, Heras M, Hermenegildo C, Medina P (2013) Aging enhances contraction to thromboxane A2 in aorta from female senescence-accelerated mice. AGE 35:117–128. 10.1007/S11357-011-9337-Y 22102320 10.1007/s11357-011-9337-y PMC3543741

[23] Nicholson CJ, Xing Y, Lee S, Liang S, Mohan S, O’Rourke C, Kang J, Morgan KG (2022) Ageing causes an aortic contractile dysfunction phenotype by targeting the expression of members of the extracellular signal-regulated kinase pathway. J Cell Mol Med 26:1456–1465. 10.1111/JCMM.17118 35181997 10.1111/jcmm.17118 PMC8899171

[24] Korzick DH, Holiman DA, Boluyt MO, Laughlin MH, Lakatta EG (2001) Diminished α1-adrenergic-mediated contraction and translocation of PKC in senescent rat heart. Am J Physiol Heart Circ Physiol 281. 10.1152/AJPHEART.2001.281.2.H581/ASSET/IMAGES/LARGE/H40810924005.JPEG 10.1152/ajpheart.2001.281.2.H581 11454560

[25] Sallam NA, Laher I (2025) Regional heterogeneity in vascular contractile dysfunction in diabetic mice. Mol Cell Biochem. 10.1007/S11010-025-05257-4 40208461 10.1007/s11010-025-05257-4

[26] Heaps CL, Bray JF, Parker JL (2020) Enhanced KCl-mediated contractility and Ca2 + sensitization in porcine collateral-dependent coronary arteries persist after exercise training. Am J Physiol Heart Circ Physiol 319:H915. 10.1152/AJPHEART.00384.2020 32857599 10.1152/ajpheart.00384.2020 PMC7654662

[27] Banerjee D, Sabe SA, Cioffi WG, Miner TJ, Sodha NR, Abid MR, Feng J, Sellke FW (2025) Age-specific increase in vasopressin-induced coronary microvascular contractile response in patients undergoing cardiac surgery. Ann Surg. 10.1097/SLA.0000000000006797 40525297 10.1097/SLA.0000000000006797

[28] Marín J, Rodríguez-Martínez MA (1999) Age-related changes in vascular responses. Exp Gerontol 34:503–512. 10.1016/S0531-5565(99)00029-7 10817806 10.1016/s0531-5565(99)00029-7

[29] Rice KM, Walker EM, Kakarla SK, Paturi S, Wu M, Narula S, Blough ER (2010) Fluprostenol-induced MAPK signaling is independent of aging in Fischer 344/NNiaHSd x Brown Norway/BiNia rat aorta - PubMed. Ann Clin Lab Sci 40:26–31 20124327

[30] Li Y, You L, Nepovimova E, Adam V, Heger Z, Jomova K, Valko M, Wu Q, Kuca K (2024) c-Jun N-terminal kinase signaling in aging. Front Aging Neurosci 16:1453710. 10.3389/FNAGI.2024.1453710 39267721 10.3389/fnagi.2024.1453710 PMC11390425

[31] Papaconstantinou J (2019) The role of signaling pathways of inflammation and oxidative stress in development of senescence and aging phenotypes in cardiovascular disease. Cells 8:1383. 10.3390/CELLS8111383 31689891 10.3390/cells8111383 PMC6912541

[32] Hao W, Shan W, Wan F, Luo J, Niu Y, Zhou J, Zhang Y, Xu N, Xie W (2023) Canagliflozin delays aging of HUVECs induced by palmitic acid via the ROS/p38/JNK pathway. Antioxidants 12:838. 10.3390/ANTIOX12040838/S1 37107212 10.3390/antiox12040838 PMC10135379

[33] Morgan D, Berggren KL, Spiess CD, Smith HM, Tejwani A, Weir SJ, Lominska CE, Thomas SM, Gan GN (2021) Mitogen-activated protein kinase-activated protein kinase-2 (MK2) and its role in cell survival, inflammatory signaling, and migration in promoting cancer. Mol Carcinog 61:173. 10.1002/MC.23348 34559922 10.1002/mc.23348 PMC8799529

[34] Li DQ, Luo L, Chen Z, Kim HS, Song XJ, Pflugfelder SC (2006) JNK and ERK MAP kinases mediate induction of IL-1β, TNF-α and IL-8 following hyperosmolar stress in human limbal epithelial cells. Exp Eye Res 82:588–596. 10.1016/J.EXER.2005.08.019 16202406 10.1016/j.exer.2005.08.019 PMC2198933

[35] Ha J, Kang E, Seo J, Cho S (2019) Phosphorylation dynamics of JNK signaling: effects of dual-specificity phosphatases (DUSPs) on the JNK pathway. Int J Mol Sci 20:6157. 10.3390/IJMS20246157 31817617 10.3390/ijms20246157 PMC6941053

[36] Bayat H, Xu S, Pimentel D, Cohen RA, Jiang B (2008) Activation of thromboxane receptor upregulates interleukin (IL)-1β-induced VCAM-1 expression through JNK signaling. Arterioscler Thromb Vasc Biol 28:127–134. 10.1161/ATVBAHA.107.150250 18032781 10.1161/ATVBAHA.107.150250

[37] Jagavelu K, Tietge UJF, Gaestel M, Drexler H, Schieffer B, Bavendiek U (2007) Systemic deficiency of the MAP kinase-activated protein kinase 2 reduces atherosclerosis in hypercholesterolemic mice. Circ Res 101:1104–1112. 10.1161/CIRCRESAHA.107.156075 17885219 10.1161/CIRCRESAHA.107.156075

[38] Winzen R, Kracht M, Ritter B, Wilhelm A, Chen CYA, Shyu AB, Müller M, Gaestel M, Resch K, Holtmann H (1999) The p38 MAP kinase pathway signals for cytokine-induced mRNA stabilization via MAP kinase-activated protein kinase 2 and an AU-rich region-targeted mechanism. EMBO J 18:4969. 10.1093/EMBOJ/18.18.4969 10487749 10.1093/emboj/18.18.4969 PMC1171568

[39] Salminen A, Kaarniranta K (2012) AMP-activated protein kinase (AMPK) controls the aging process via an integrated signaling network. Ageing Res Rev 11:230–241. 10.1016/j.arr.2011.12.005 22186033 10.1016/j.arr.2011.12.005

[40] Shi D, Mi G, Wang M, Webster TJ (2018) In vitro and ex vivo systems at the forefront of infection modeling and drug discovery. Biomaterials 198:228. 10.1016/J.BIOMATERIALS.2018.10.030 30384974 10.1016/j.biomaterials.2018.10.030 PMC7172914

[41] Gorgulu S, Eren M, Celik S, Dagdeviren B, Uslu N, Suer N, Tezel T (2003) The effects of hormonal therapy on aortic stiffness and left ventricular diastolic function. Acta Cardiol 58:1–8. 10.2143/AC.58.1.2005252 12625488 10.2143/AC.58.1.2005252

[42] Mu W, Chen M, Gong Z, Zheng F, Xing Q (2015) Expression of vascular cell adhesion molecule-1 in the aortic tissues of atherosclerotic patients and the associated clinical implications. Exp Ther Med 10:423. 10.3892/ETM.2015.2540 26622332 10.3892/etm.2015.2540 PMC4509110

[43] Sun Y, Byon CH, Yang Y, Bradley WE, Dell’Italia LJ, Sanders PW, Agarwal A, Wu H, Chen Y (2017) Dietary potassium regulates vascular calcification and arterial stiffness. JCI Insight 2:e94920. 10.1172/JCI.INSIGHT.94920 28978809 10.1172/jci.insight.94920 PMC5841863

[44] Cheung-Flynn J, Alvis BD, Hocking KM, Guth CM, Luo W, McCallister R, Chadalavada K, Polcz M, Komalavilas P, Brophy CM (2019) Normal saline solutions cause endothelial dysfunction through loss of membrane integrity, ATP release, and inflammatory responses mediated by P2X7R/p38 MAPK/MK2 signaling pathways. PLoS One 14:e0220893. 10.1371/JOURNAL.PONE.0220893 31412063 10.1371/journal.pone.0220893 PMC6693757

[45] Orr AW, Hastings NE, Blackman BR, Wamhoff BR (2009) Complex regulation and function of the inflammatory smooth muscle cell phenotype in atherosclerosis. J Vasc Res 47:168. 10.1159/000250095 19851078 10.1159/000250095 PMC2842170

[46] Lee HNR, Lin J, Smith CJ, Ware LB, Harrison FE, Bastarache JA, Baer B (2025) Advanced age in mice exacerbates sepsis-induced inflammation, vascular permeability, and multi-organ dysfunction. Shock. 10.1097/SHK.0000000000002657 40668087 10.1097/SHK.0000000000002657 PMC12838851

[47] Zhang S, Xia B, Kalionis B, Li H, Zhang X, Zhang X, Xia S (2024) The role and mechanism of vascular aging in geriatric vascular diseases. Aging Dis 16:2237–2236. 10.14336/AD.2024.0717 39325934 10.14336/AD.2024.0717 PMC12221404

[48] Donato AJ, Machin DR, Lesniewski LA (2018) Mechanisms of dysfunction in the aging vasculature and role in age-related disease. Circ Res 123:825–848. 10.1161/CIRCRESAHA.118.312563 30355078 10.1161/CIRCRESAHA.118.312563 PMC6207260

[49] Han Y, Kim SY (2023) Endothelial senescence in vascular diseases: current understanding and future opportunities in senotherapeutics. Exp Mol Med 55:1–12. 10.1038/S12276-022-00906-W 36599934 10.1038/s12276-022-00906-w PMC9898542

[50] Diana P, Carvalheira GMG (2022) NIBAN1, exploring its roles in cell survival under stress context. Front Cell Dev Biol. 10.3389/FCELL.2022.867003 35517496 10.3389/fcell.2022.867003 PMC9062034

[51] Ganguly K, Levänen B, Palmberg L, Åkesson A, Lindén A (2018) Cadmium in tobacco smokers: a neglected link to lung disease? Eur Respir Rev. 10.1183/16000617.0122-2017 29592863 10.1183/16000617.0122-2017 PMC9488953

[52] Ximenes CF, Rodrigues SML, Podratz PL, Merlo E, de Araújo JFP, Rodrigues LCM, Coitinho JB, Vassallo DV, Graceli JB, Stefanon I (2017) Tributyltin chloride disrupts aortic vascular reactivity and increases reactive oxygen species production in female rats. Environ Sci Pollut Res Int 24:24509–24520. 10.1007/S11356-017-0061-8 28900851 10.1007/s11356-017-0061-8

[53] Cary CM, Seymore TN, Singh D, Vayas KN, Goedken MJ, Adams S, Polunas M, Sunil VR, Laskin DL, Demokritou P, Stapleton PA (2023) Single inhalation exposure to polyamide micro and nanoplastic particles impairs vascular dilation without generating pulmonary inflammation in virgin female Sprague Dawley rats. Part Fibre Toxicol. 10.1186/S12989-023-00525-X 37088832 10.1186/s12989-023-00525-x PMC10122824

