# Supplementary material for: Endothelial dysfunction in aging associated with reduced Niban phosphorylation
Source: Mol Biol Rep. 2026 Jan 29;53(1):335. doi: 10.1007/s11033-026-11504-8 (PMC12855232; doi:10.1007/s11033-026-11504-8)
Supplement: Supplementary file 1 — Supplementary material 1 (DOCX 352.2 kb) [file 11033_2026_11504_MOESM1_ESM.docx]

Supplemental Figures:

**
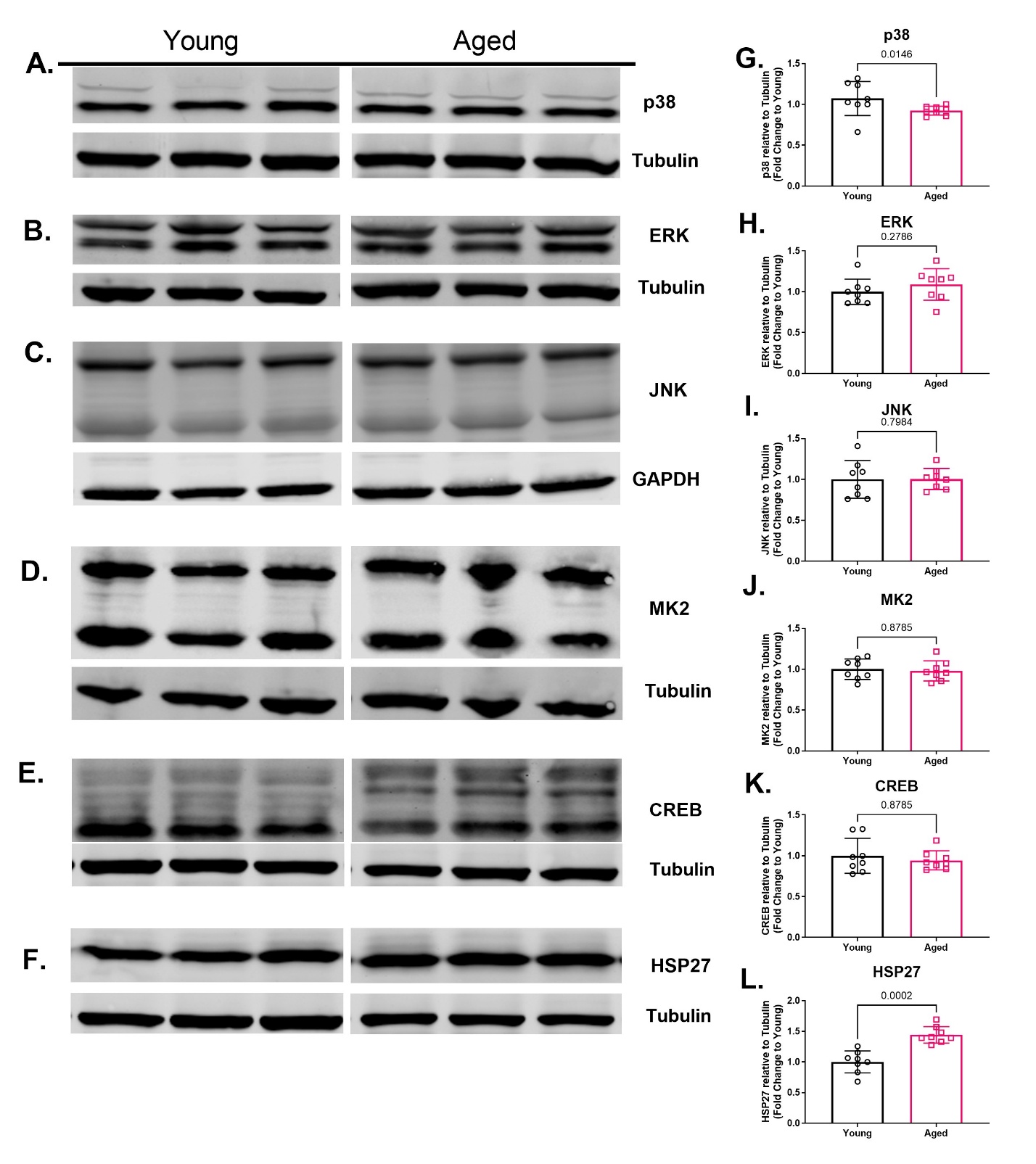
**

**Supplemental Fig. 1** Protein levels for unphosphorylated stress-activated kinases and transcripiton factors downstream of the MAPK pathways in aortas isolated from aged (20- to 23-months-old) and young (3-months-old) rats. Representative Western blots for total p38 (A), ERK (B), JNK (C), MK2 (D), CREB (E), and HSP27 (F), as well as their respective house keeping protein tubulin or GAPDH. Aged rat aortas had lower protein levels of total p38 (G) compared to young rat aortas. However, no differences were observed between young and aged rat aortas for protein levels of total ERK (H), JNK (I), MK2 (J) or CREB (K). However, aged rat aortas had higher protein levels for HSP27 (L). Data were presented as individual data points each representing an individual animal (F-J). Bars represent mean and error bars represent standard deviation. N = 8. [Statistical analysis: Mann-Whitney test (G-L)]. MAPK = mitogen-activated protein kinase, ERK = Extracellular-signal-regulated kinase, JNK = c-Jun N-terminal kinases, MK-2 = MAPK-activated protein kinase 2, CREB = cAMP response element-binding protein, HSP27 = heat shock protein 27
