# Supplementary material for: Endothelial dysfunction in aging associated with reduced Niban phosphorylation
Source: Mol Biol Rep. 2026 Jan 29;53(1):335. doi: 10.1007/s11033-026-11504-8 (PMC12855232; doi:10.1007/s11033-026-11504-8)

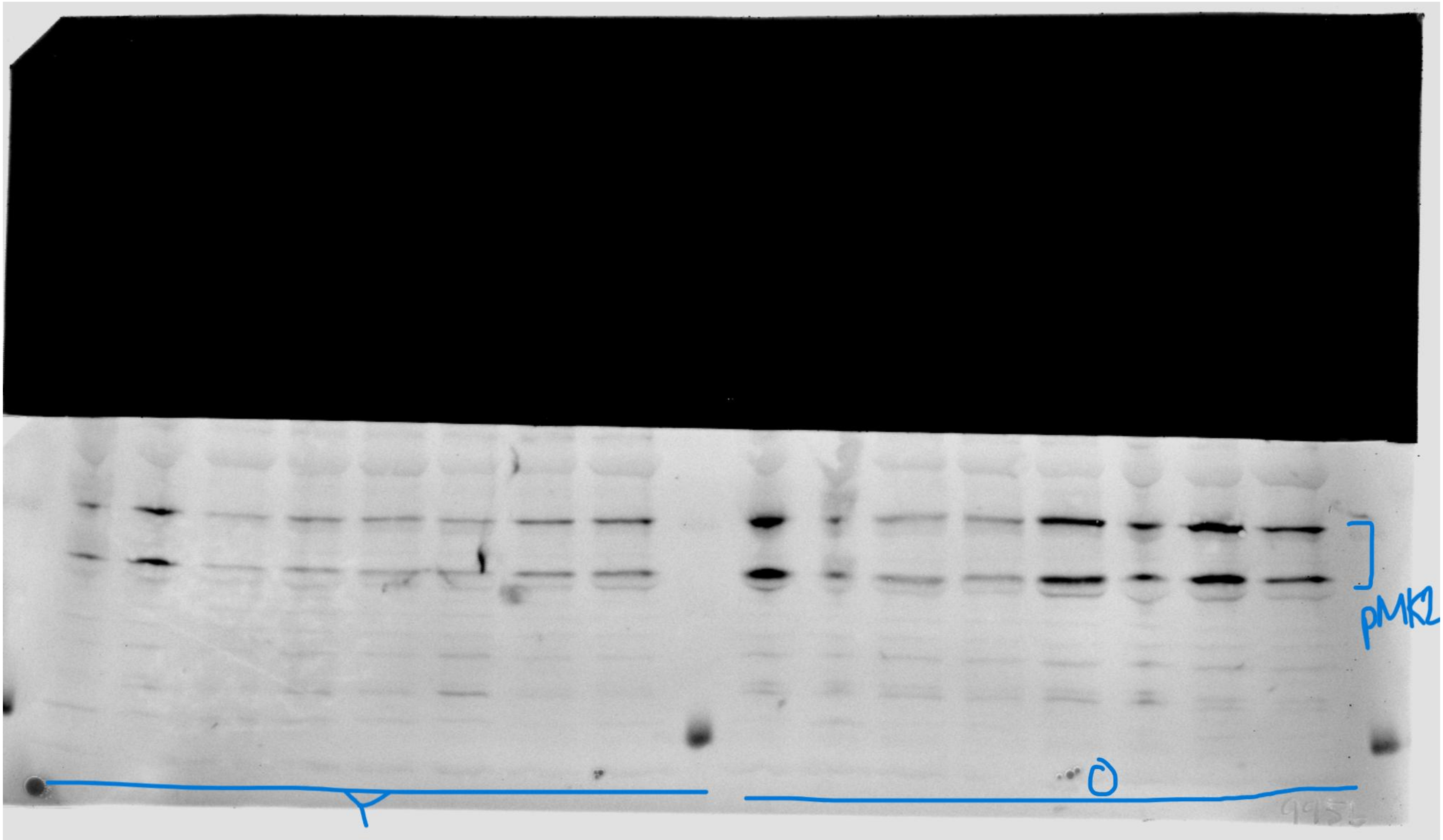

Young (3-month-old) **p-MK2** Aged (20- to 23-month-old)

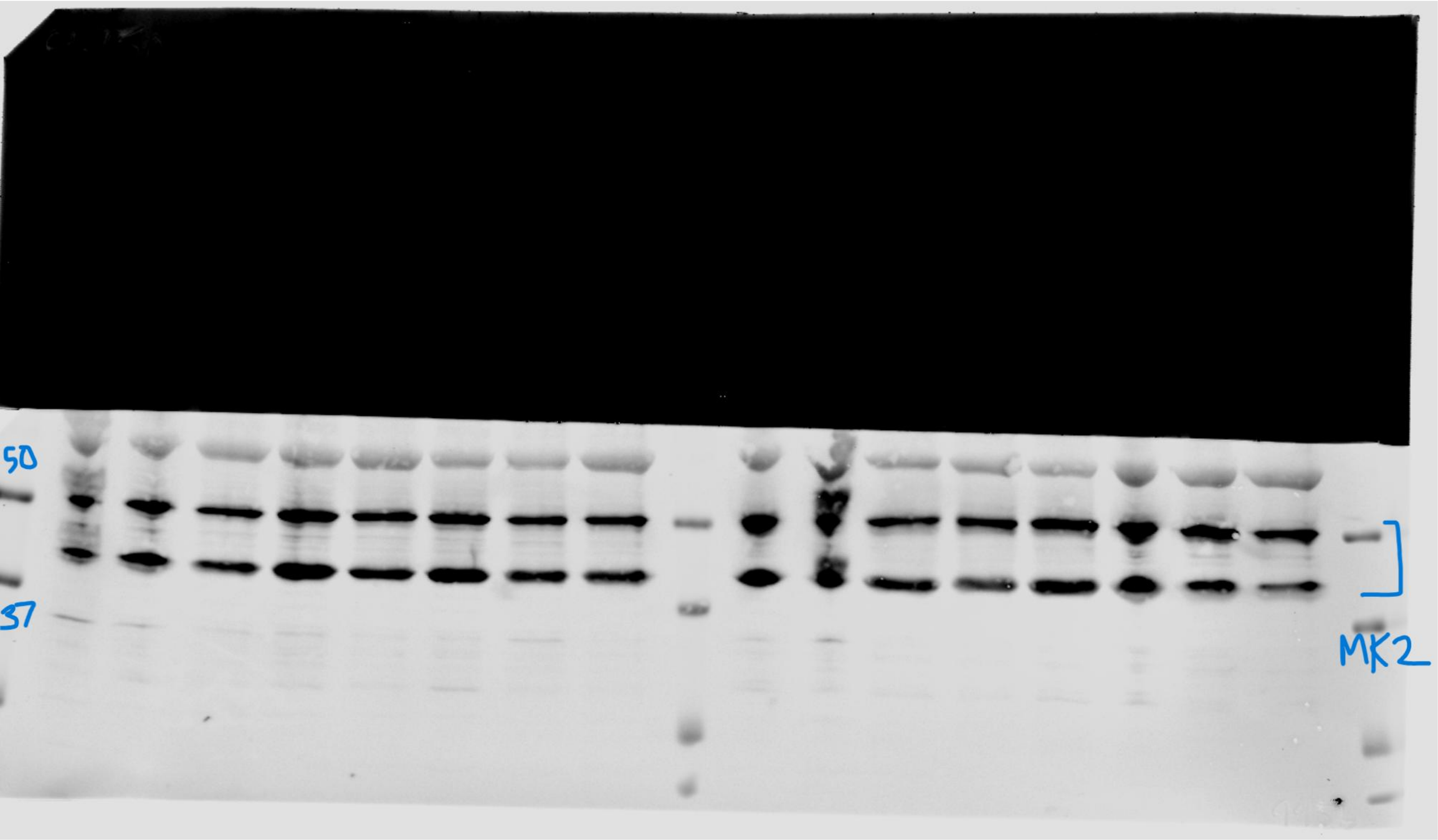

Young (3-month-old) **Total MK2** Aged (20- to 23-month-old)

Young (3-month-old)

Aged (20- to 23-month-old)

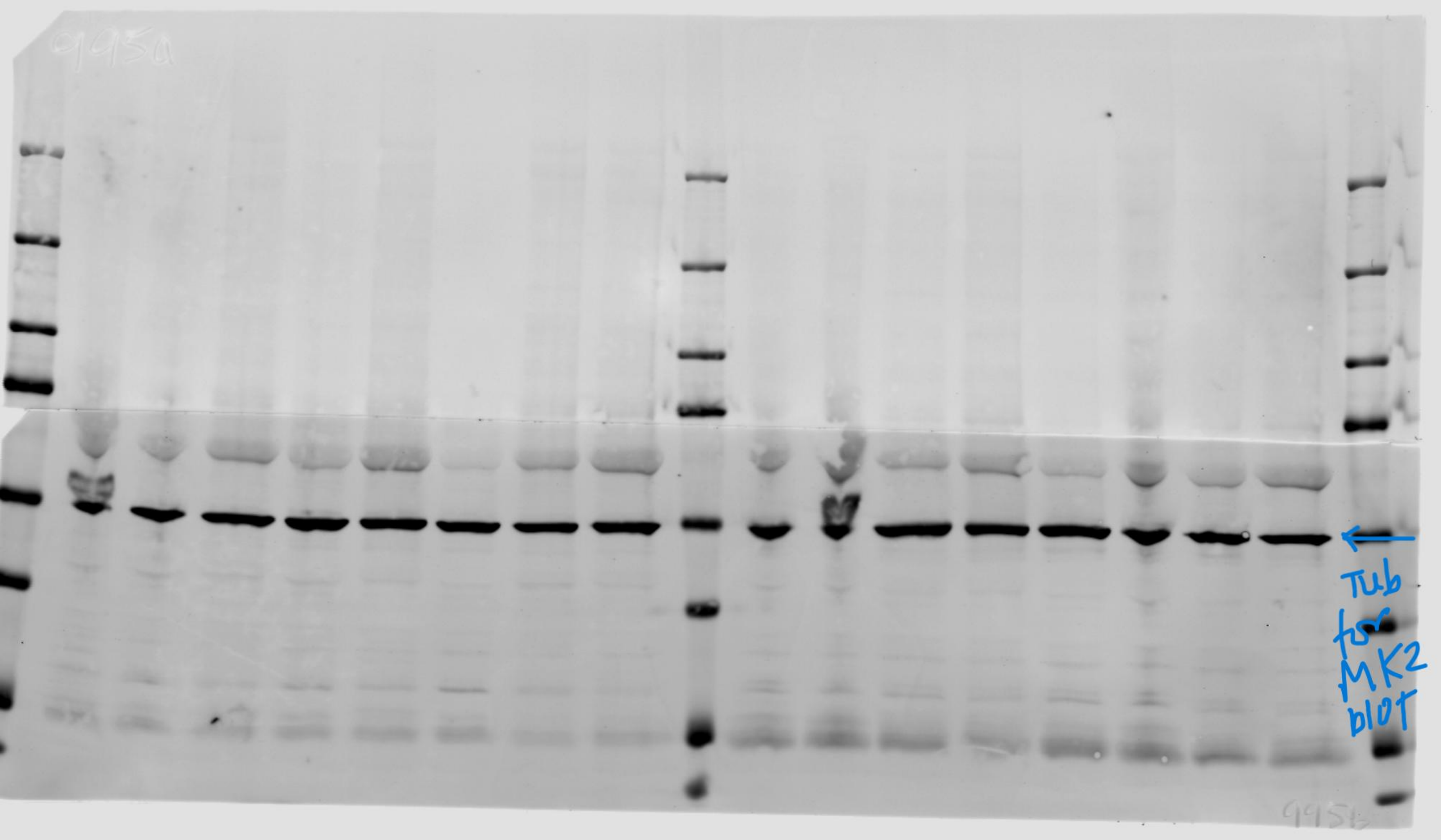

Tubulin for MK2 blot

Young (3-month-old) Aged (20- to 23-month-old)

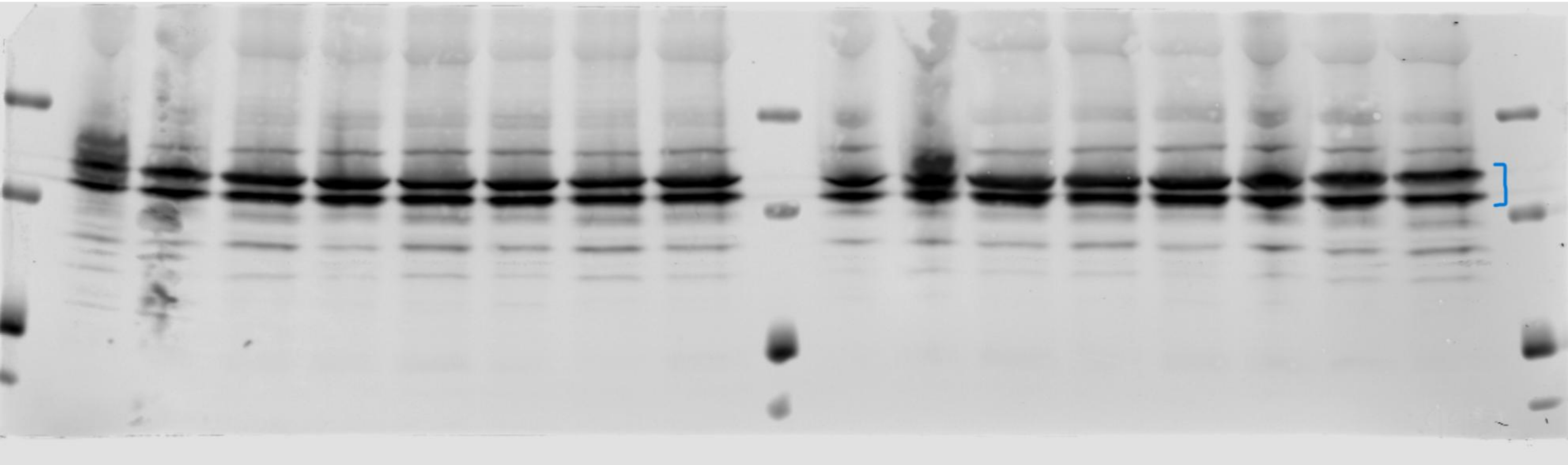

p-ERK

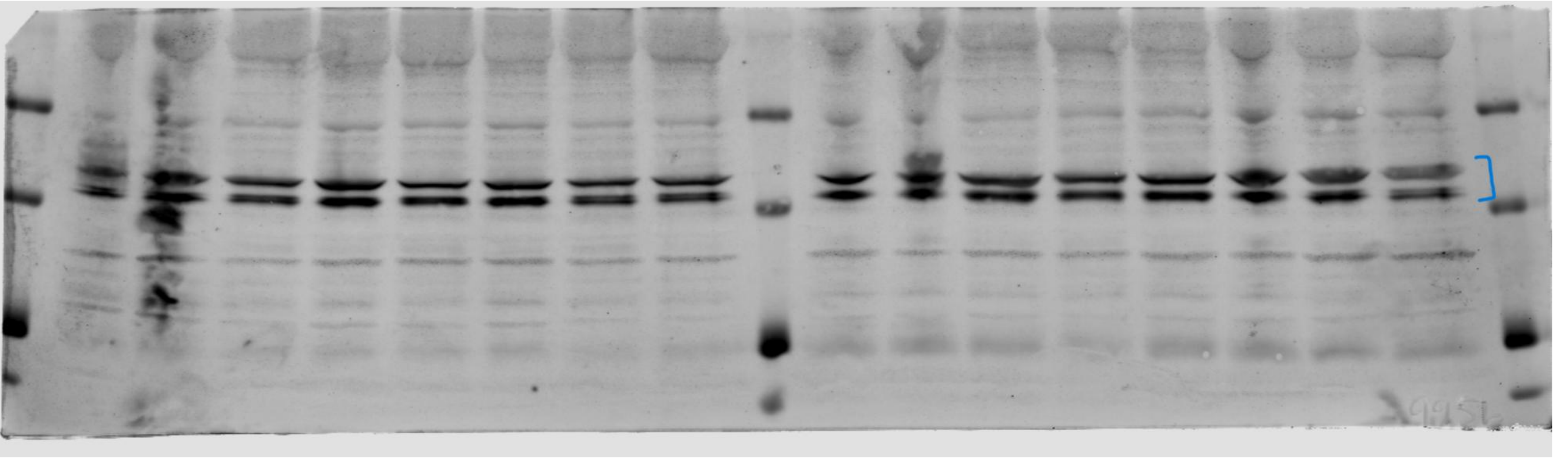

ERK

Young (3-month-old)
Aged (20- to 23-month-old)

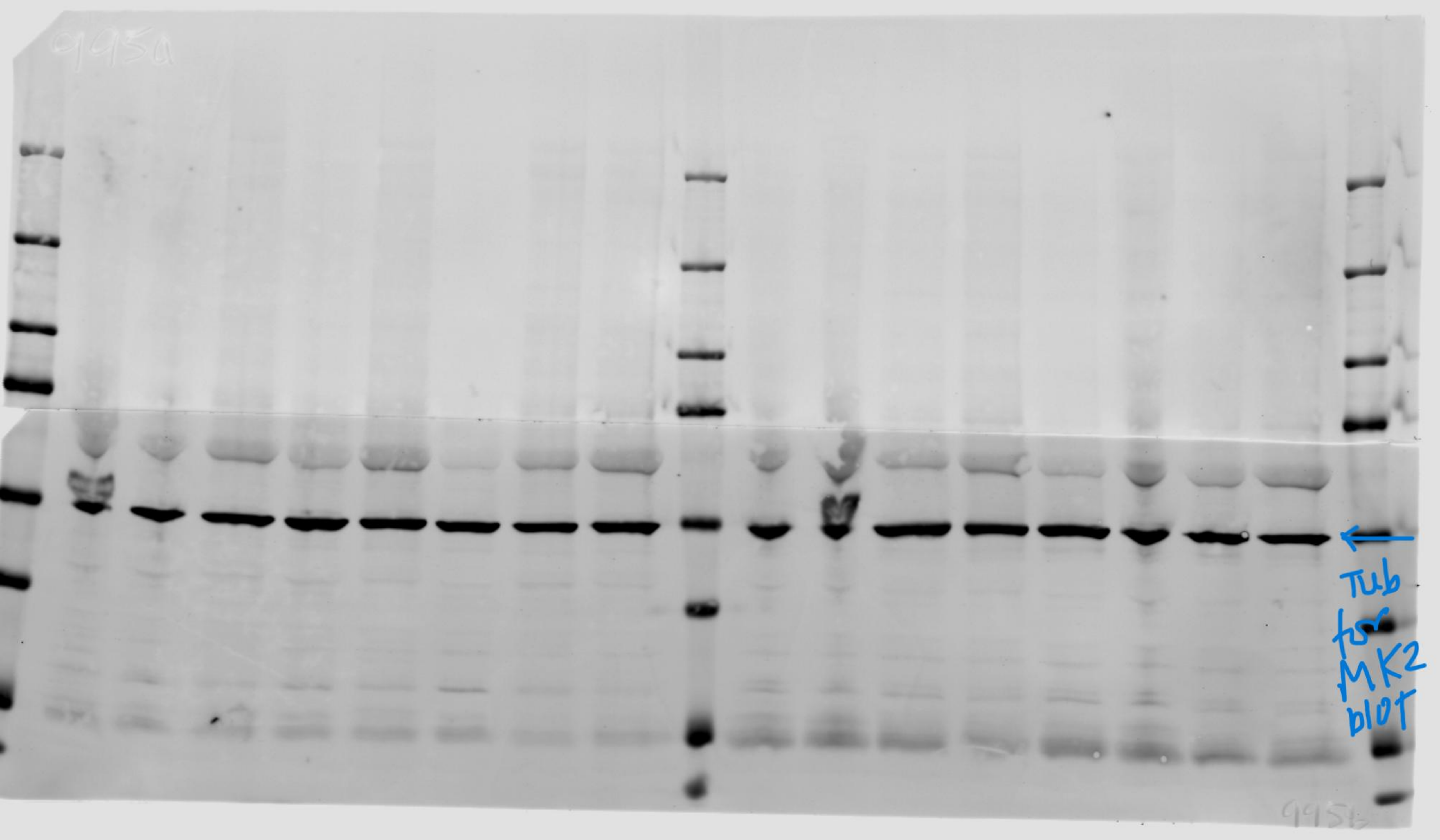

Tubulin for ERK is the same blot as MK2

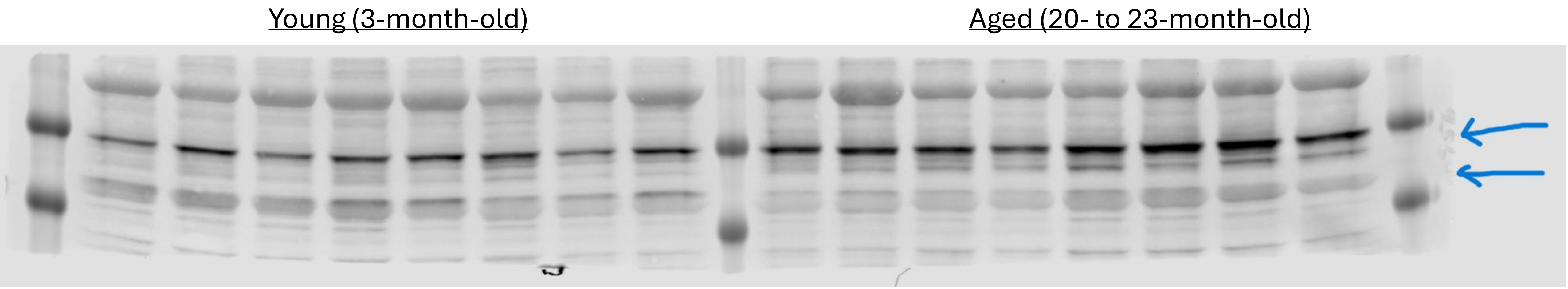

p-JNK (two bands 57 and 46.5)

Young (3-month-old) Aged (20- to 23-month-old)

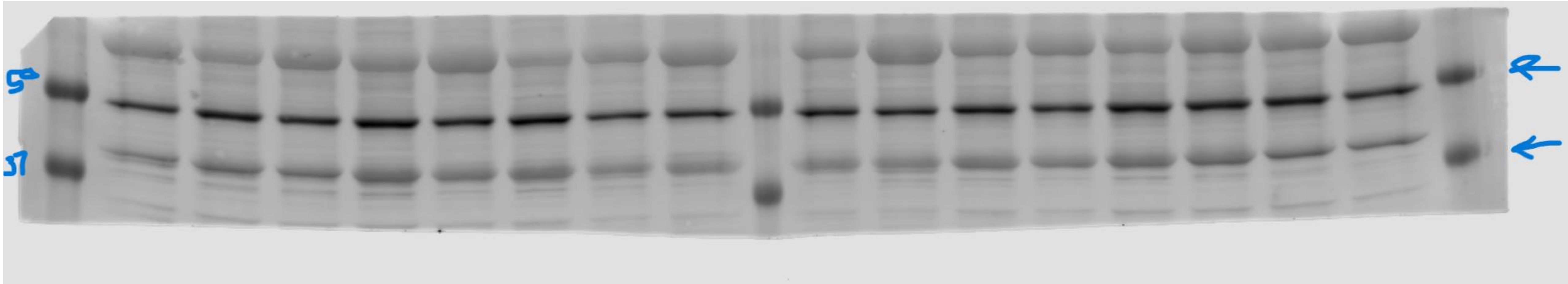

JNK (two bands 46.5, 57)

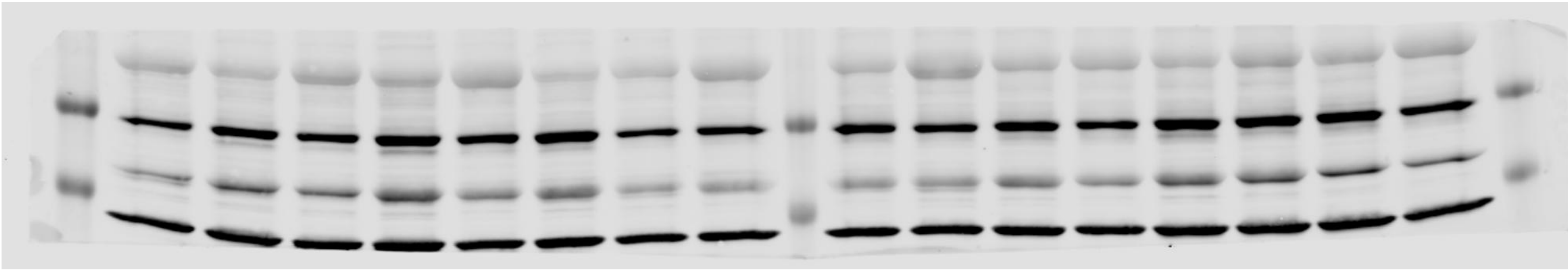

GAPDH for JNK

Young (3-month-old)　　　　　　　　　　Aged (20- to 23-month-old)

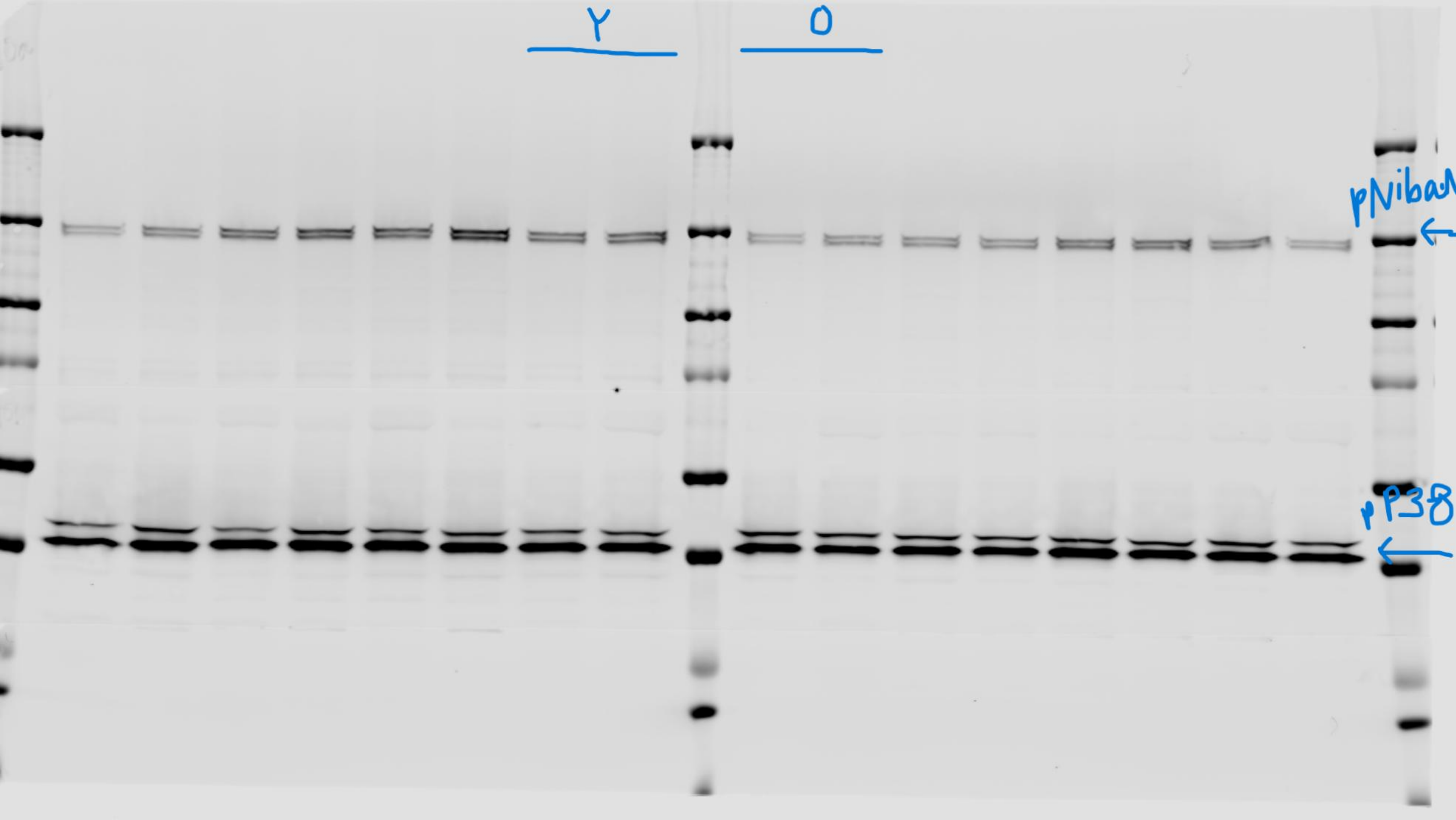

p-NIBAN and p-p38

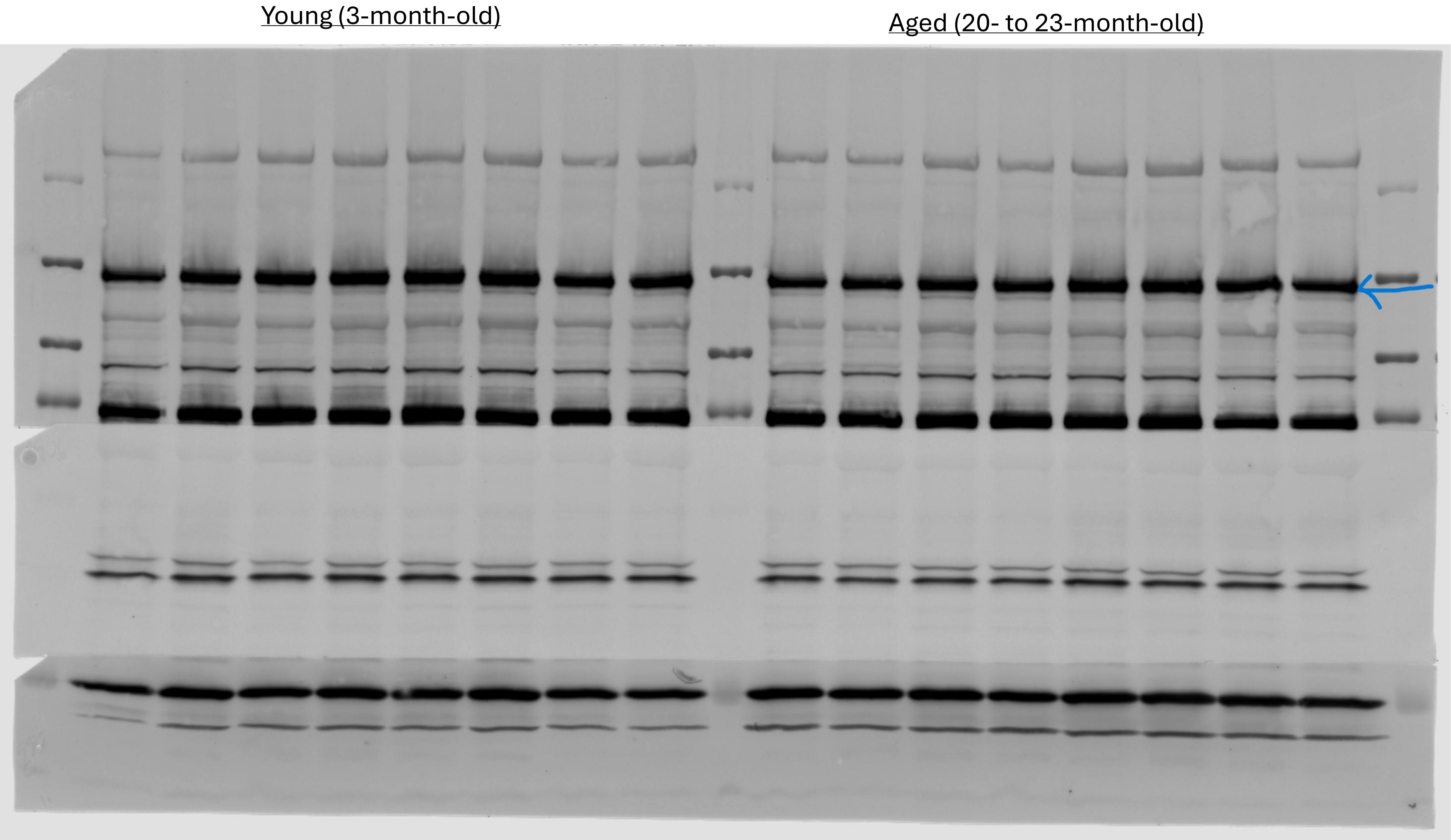

Niban (total)

Young (3-month-old)

Aged (20- to 23-month-old)

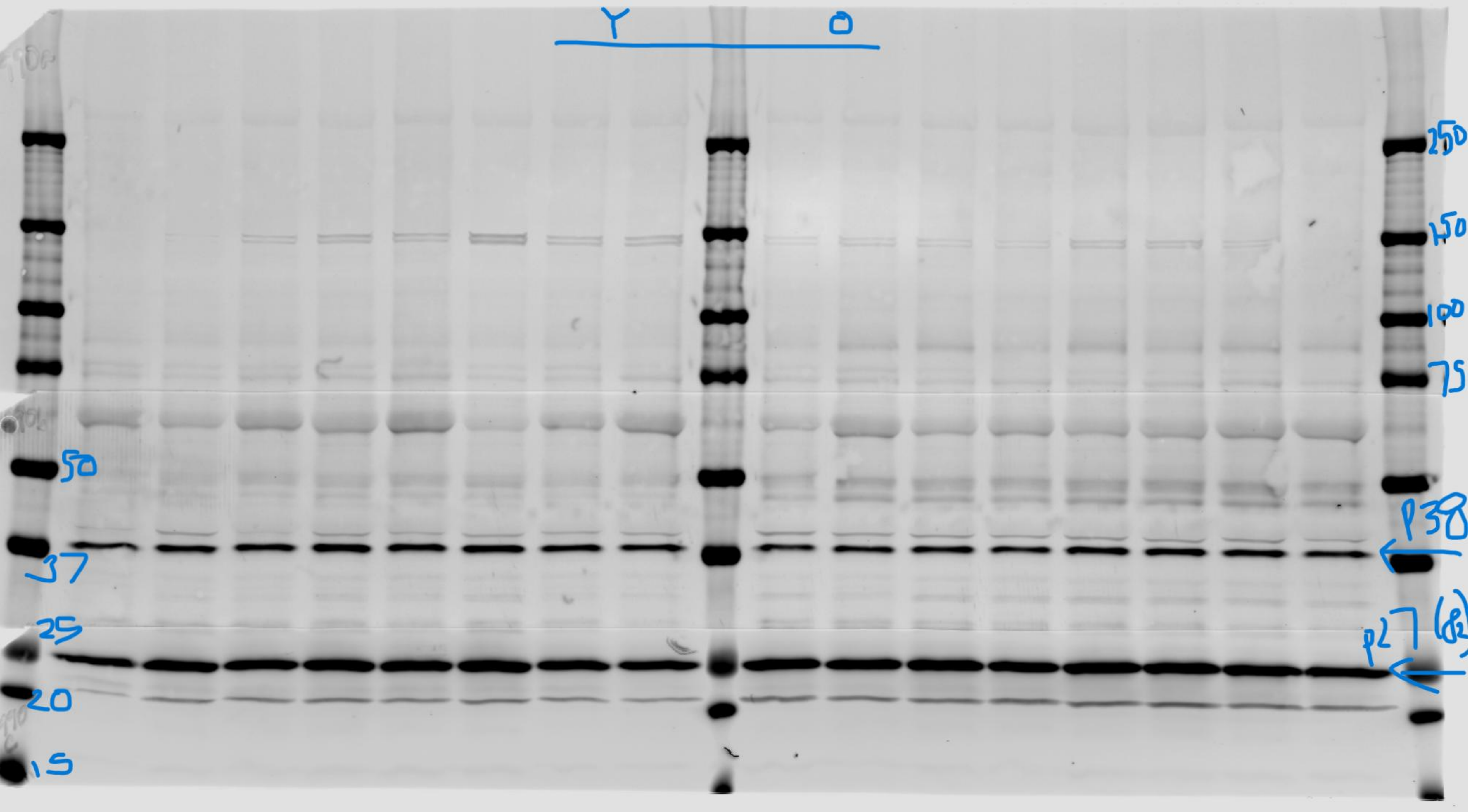

p38 total and phospho-HSP27 (serine 82)

Young (3-month-old)

Aged (20- to 23-month-old)

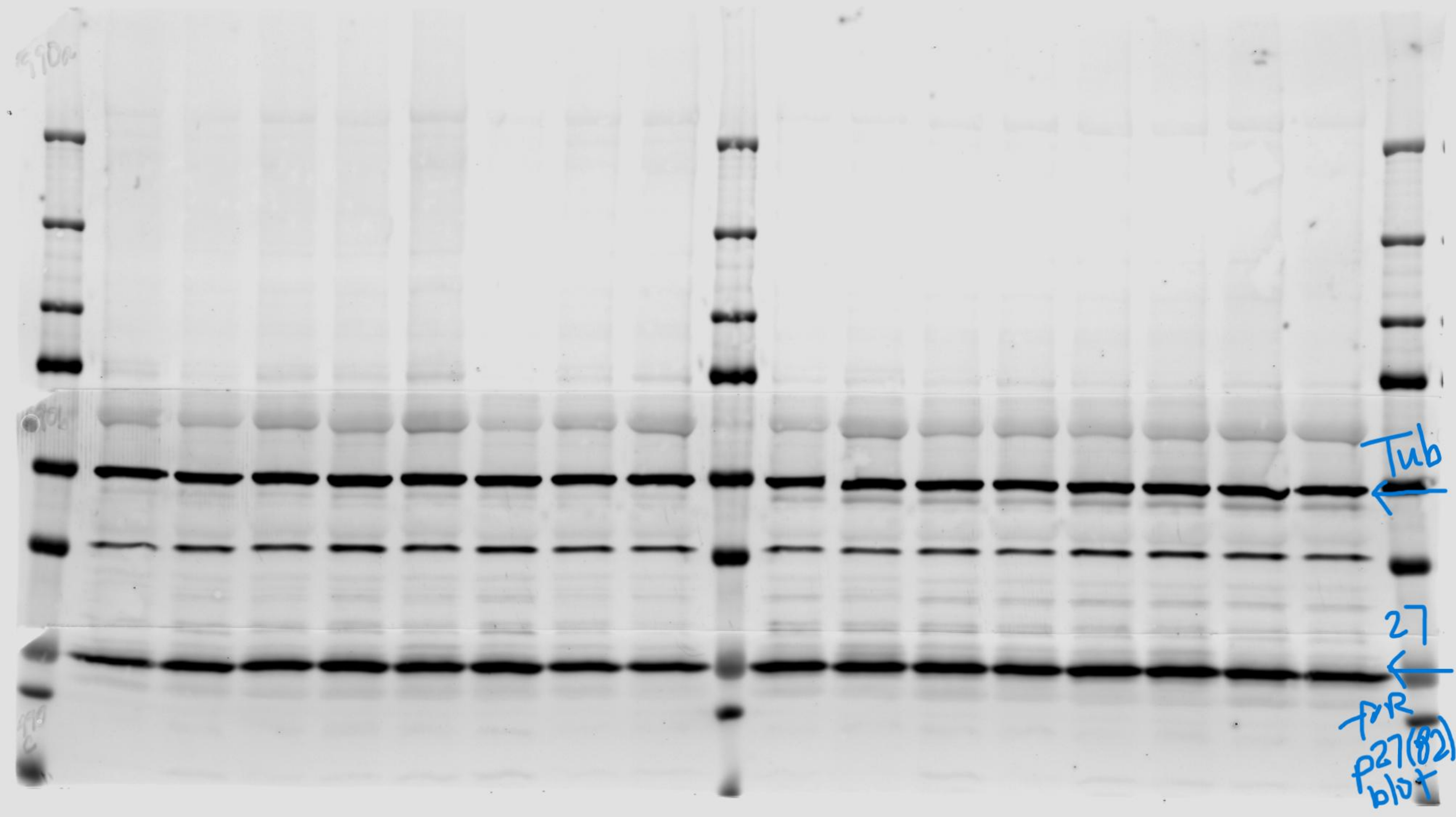

Tubulin

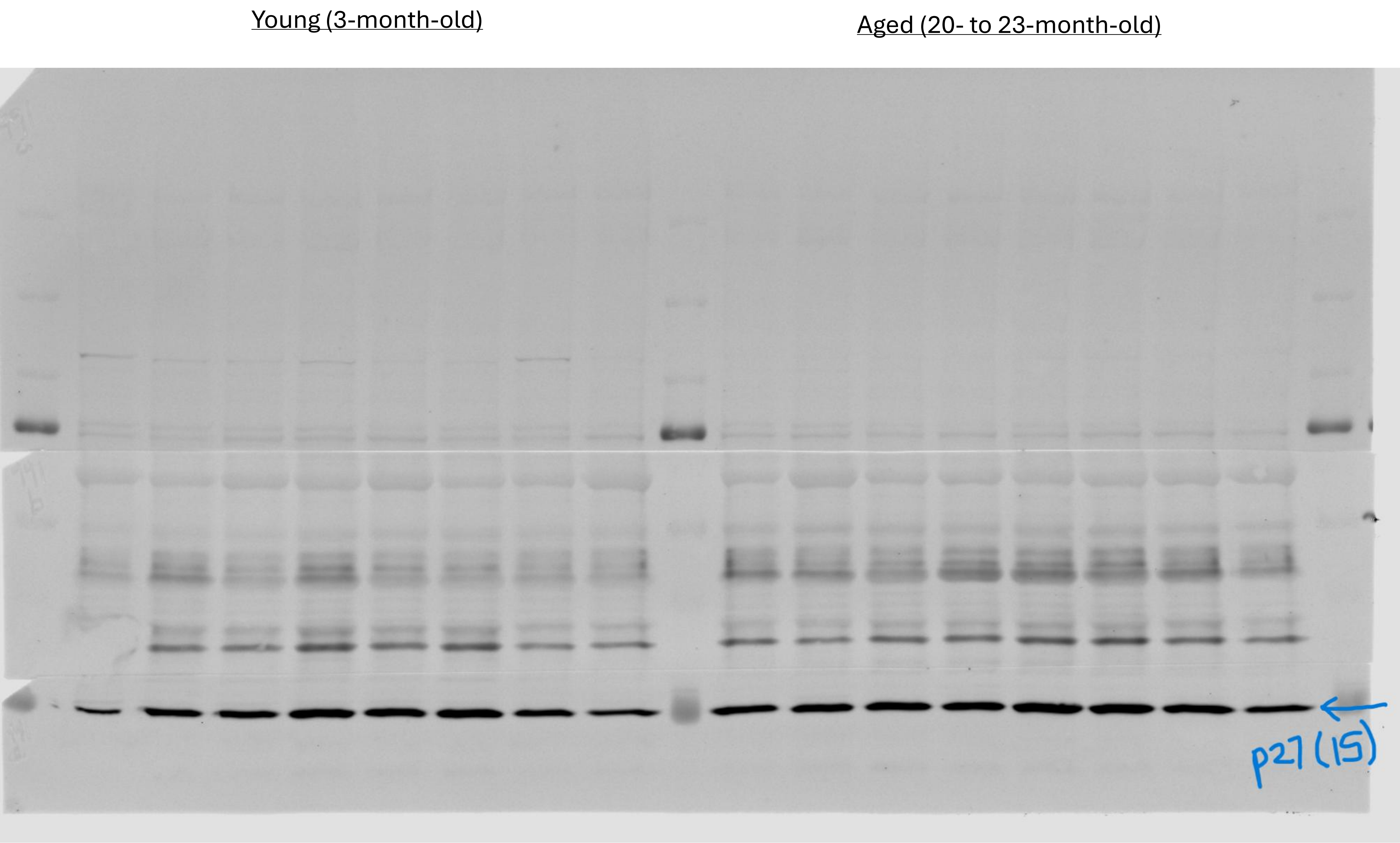

p-HSP27 (serine15)

Young (3-month-old) Aged (20- to 23-month-old)

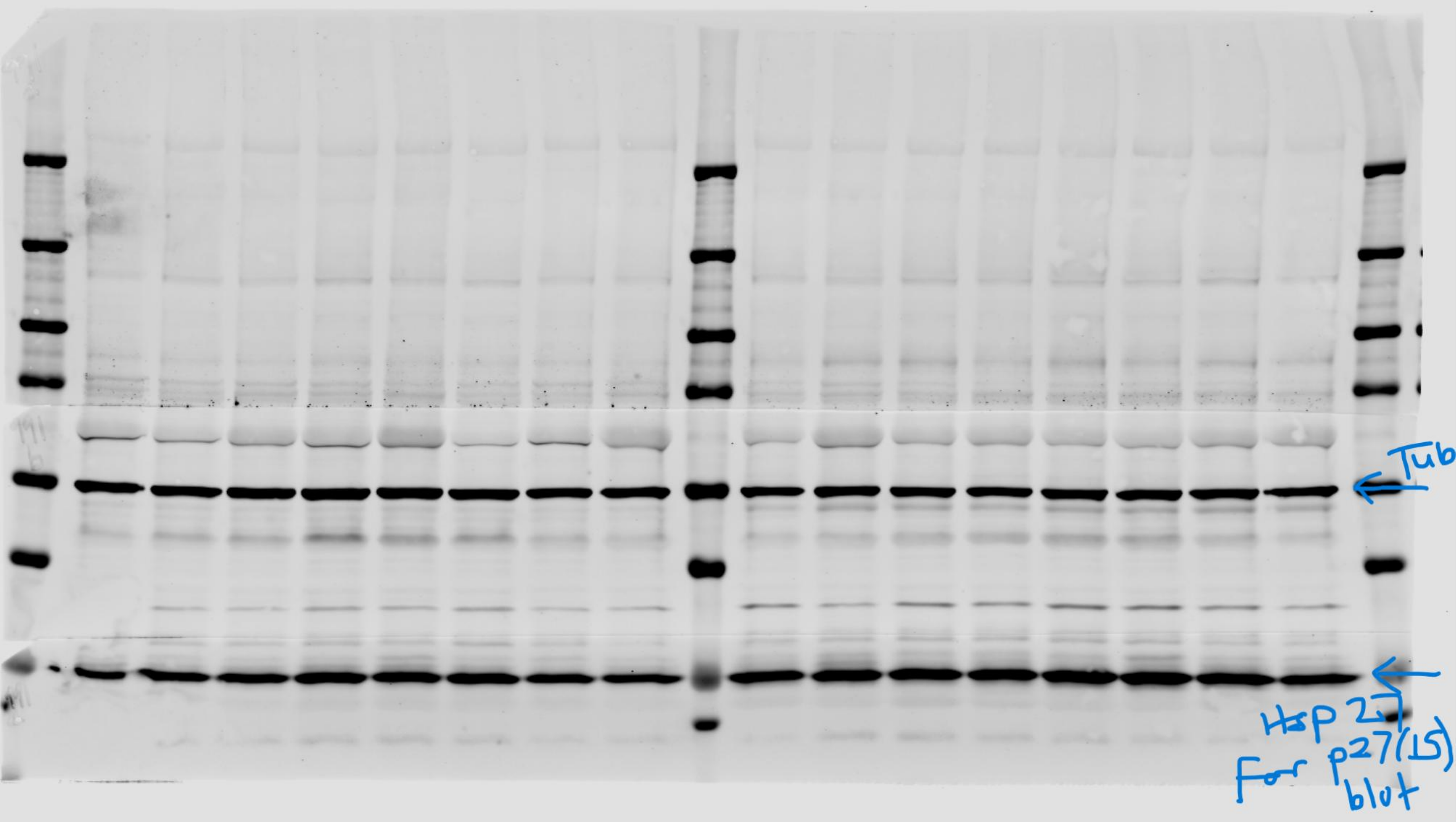

Tubulin and HSP27 for p-HSP27 (serine 15)

Young (3-month-old)

Aged (20- to 23-month-old)

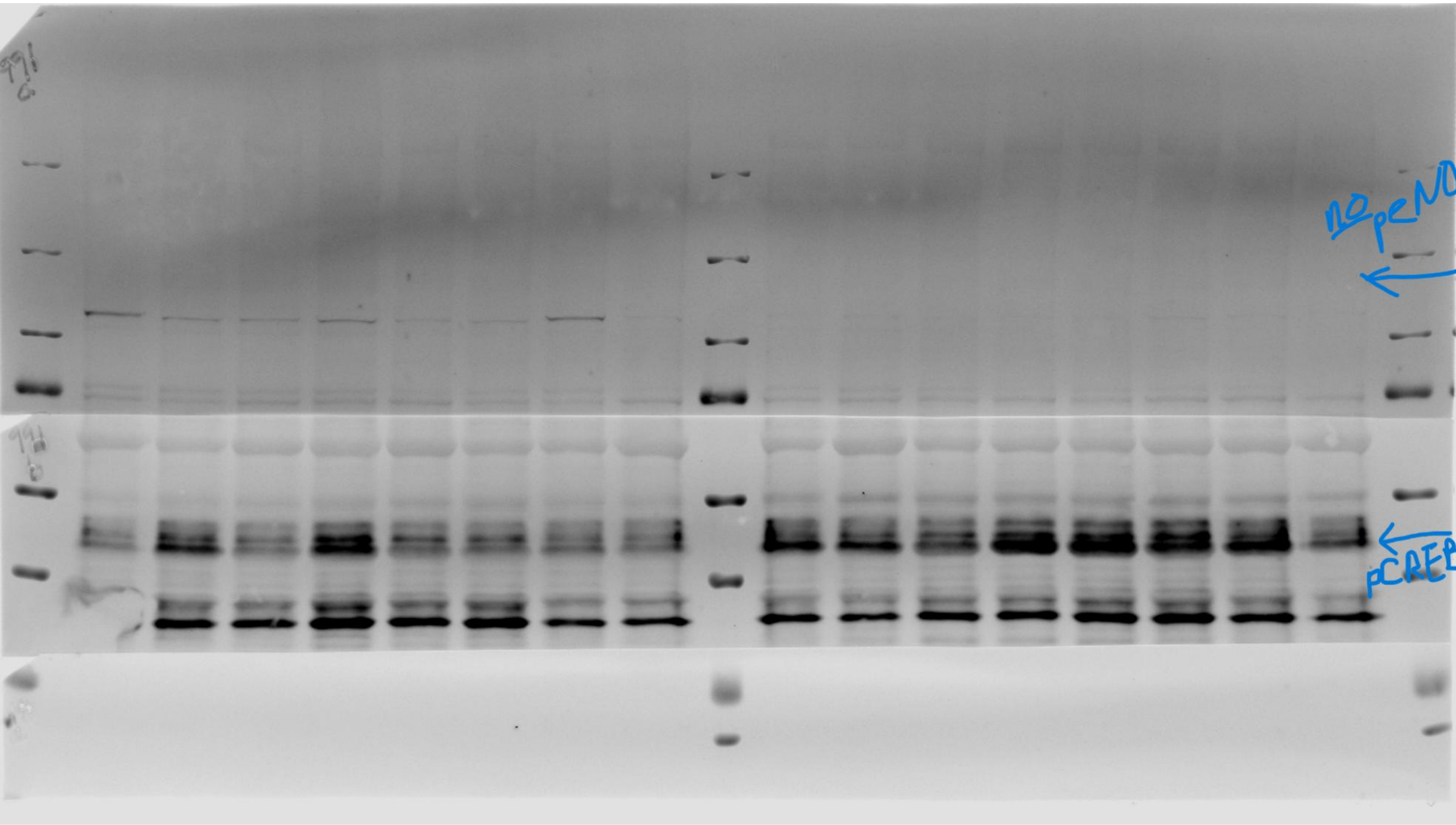

p-CREB

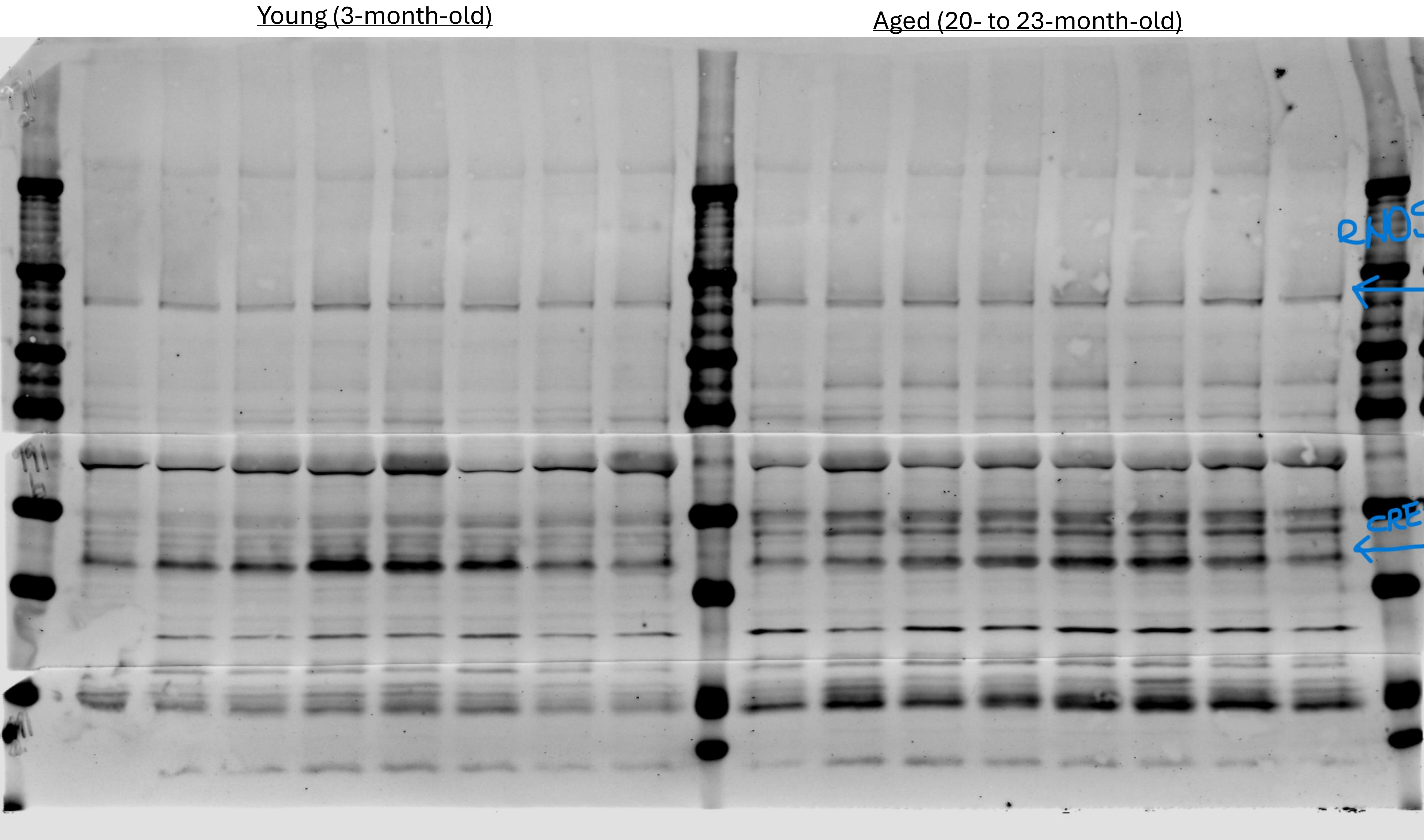

CREB total

Tubulin for CREB (Same blot as pHSP27 (15))

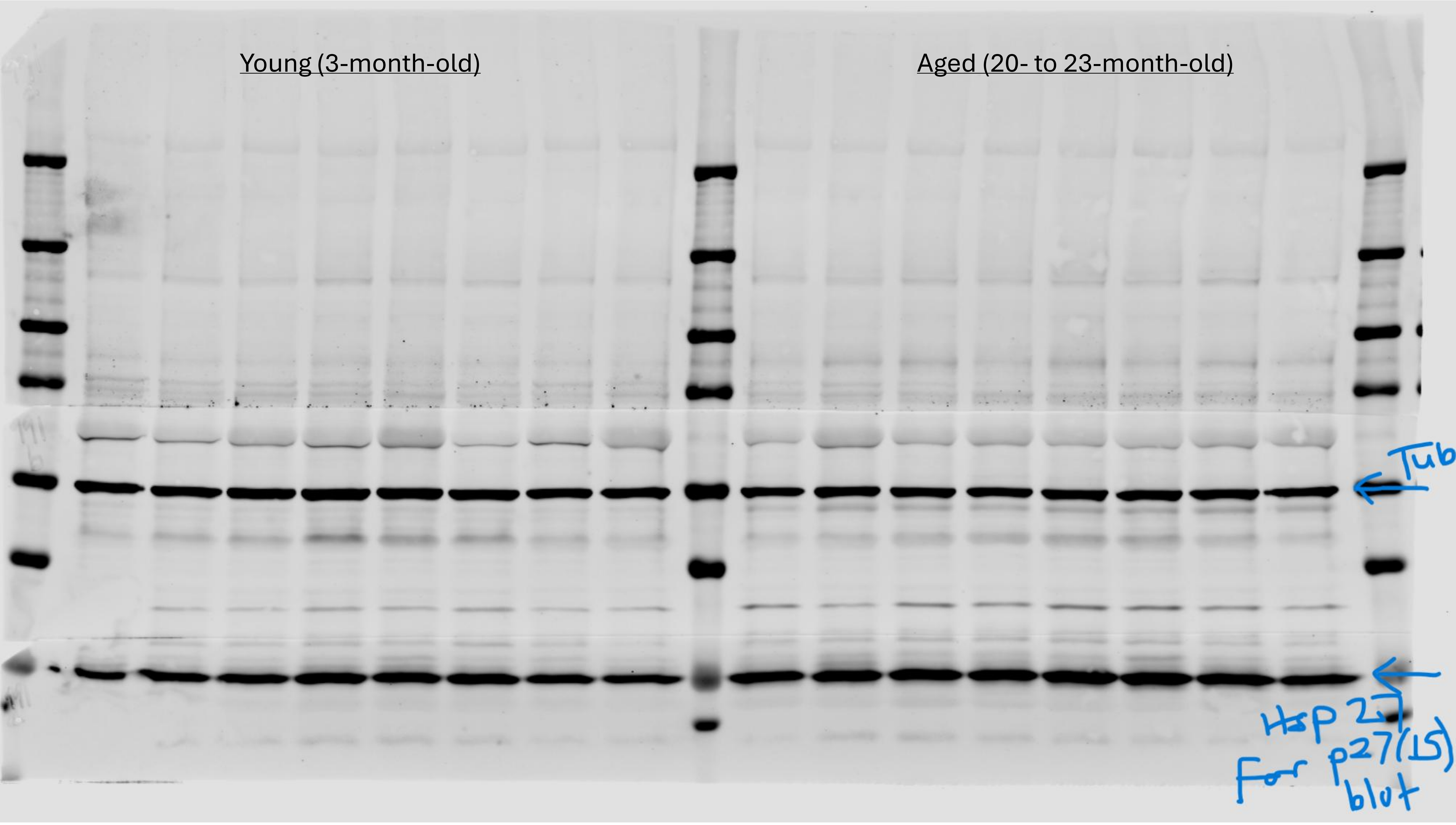

Young (3-month-old) Aged (20- to 23-month-old)

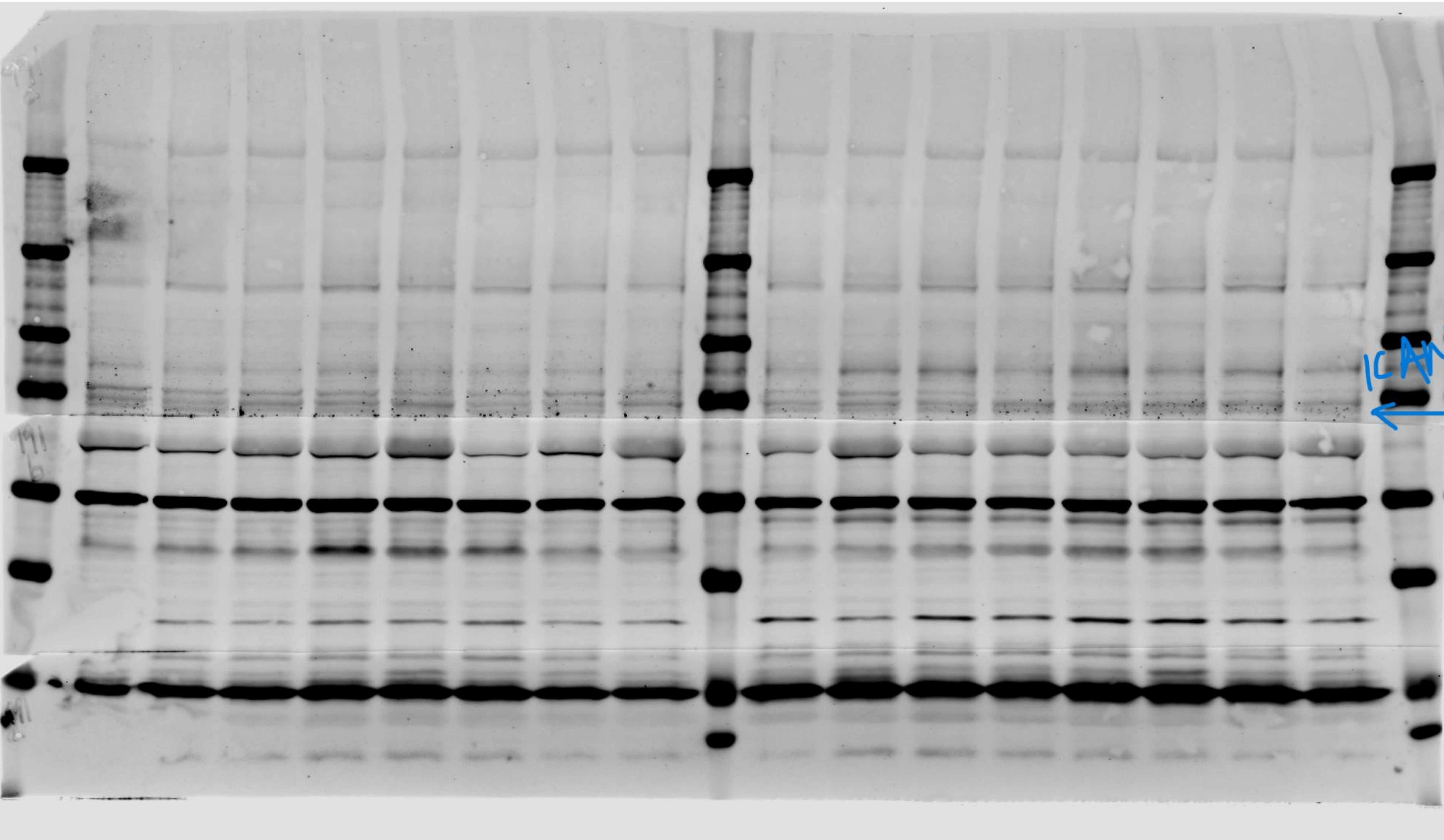

ICAM-1

Young (3-month-old) Aged (20- to 23-month-old)

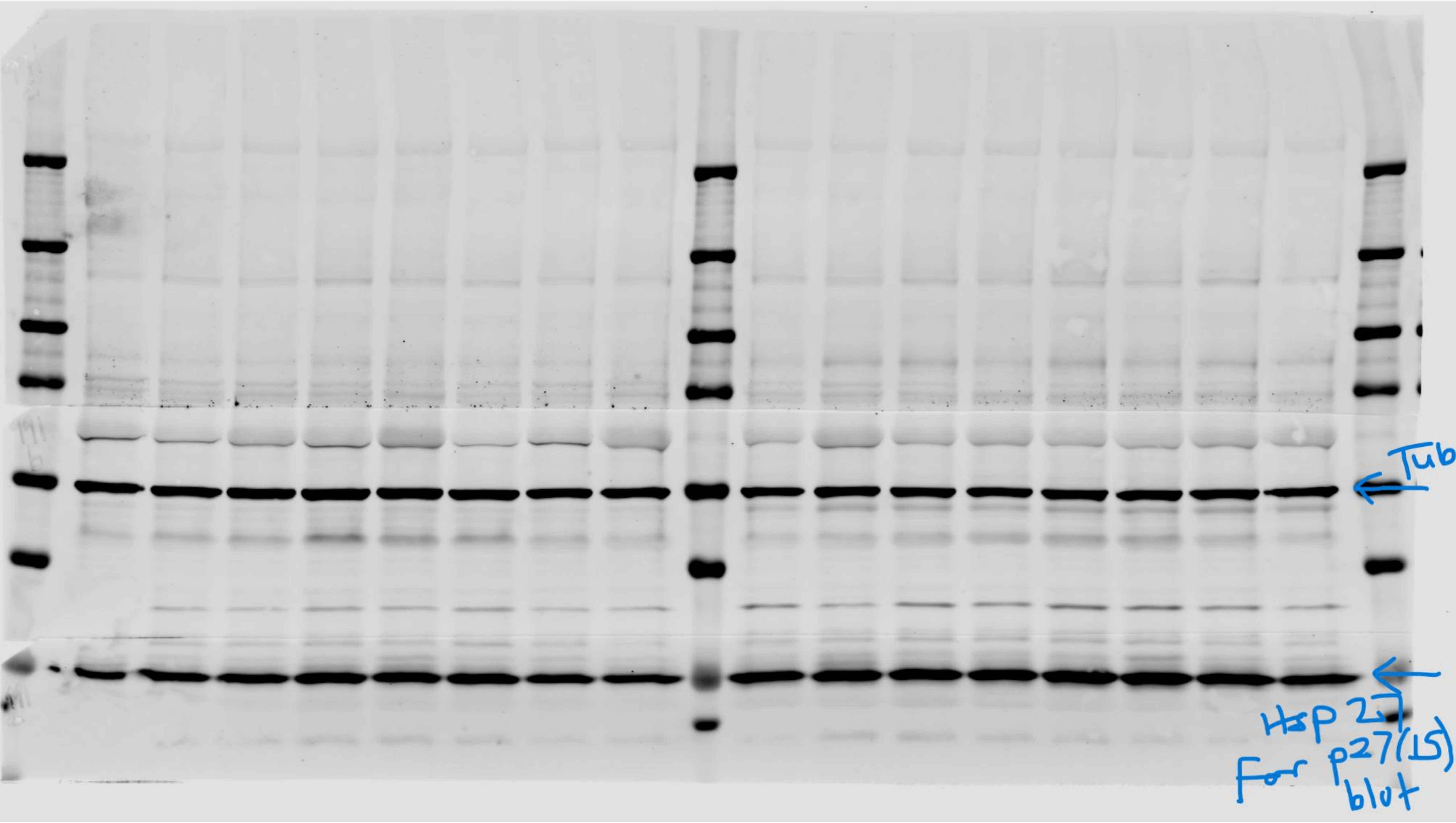

Tubulin for ICAM-1 (Same blot as pHSP27 (15))

Representative blots: Phosphorylated and Total protein

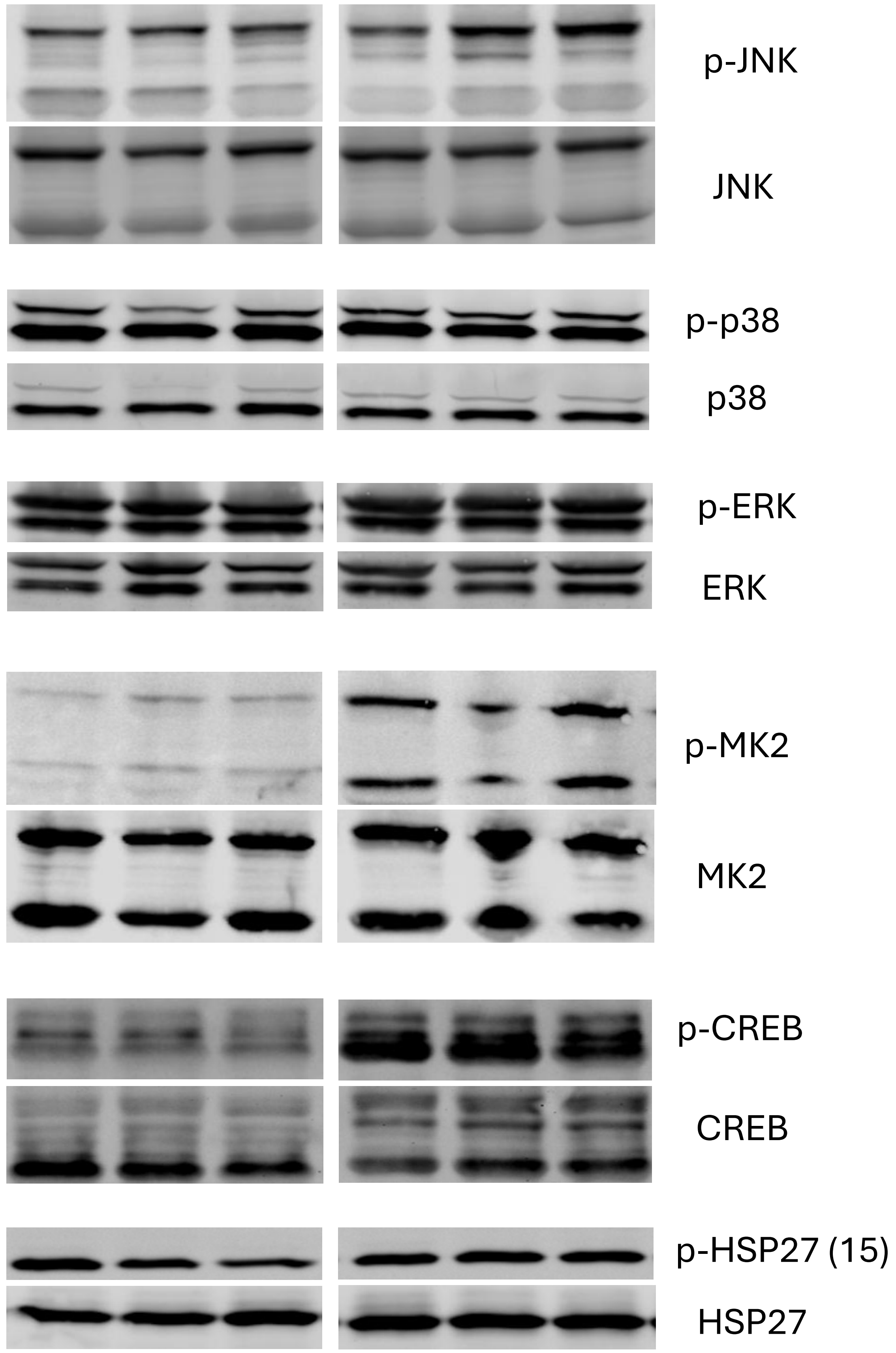

Representative Blots: Unphosphorylated (total) protein, Tubulin, and GAPDH

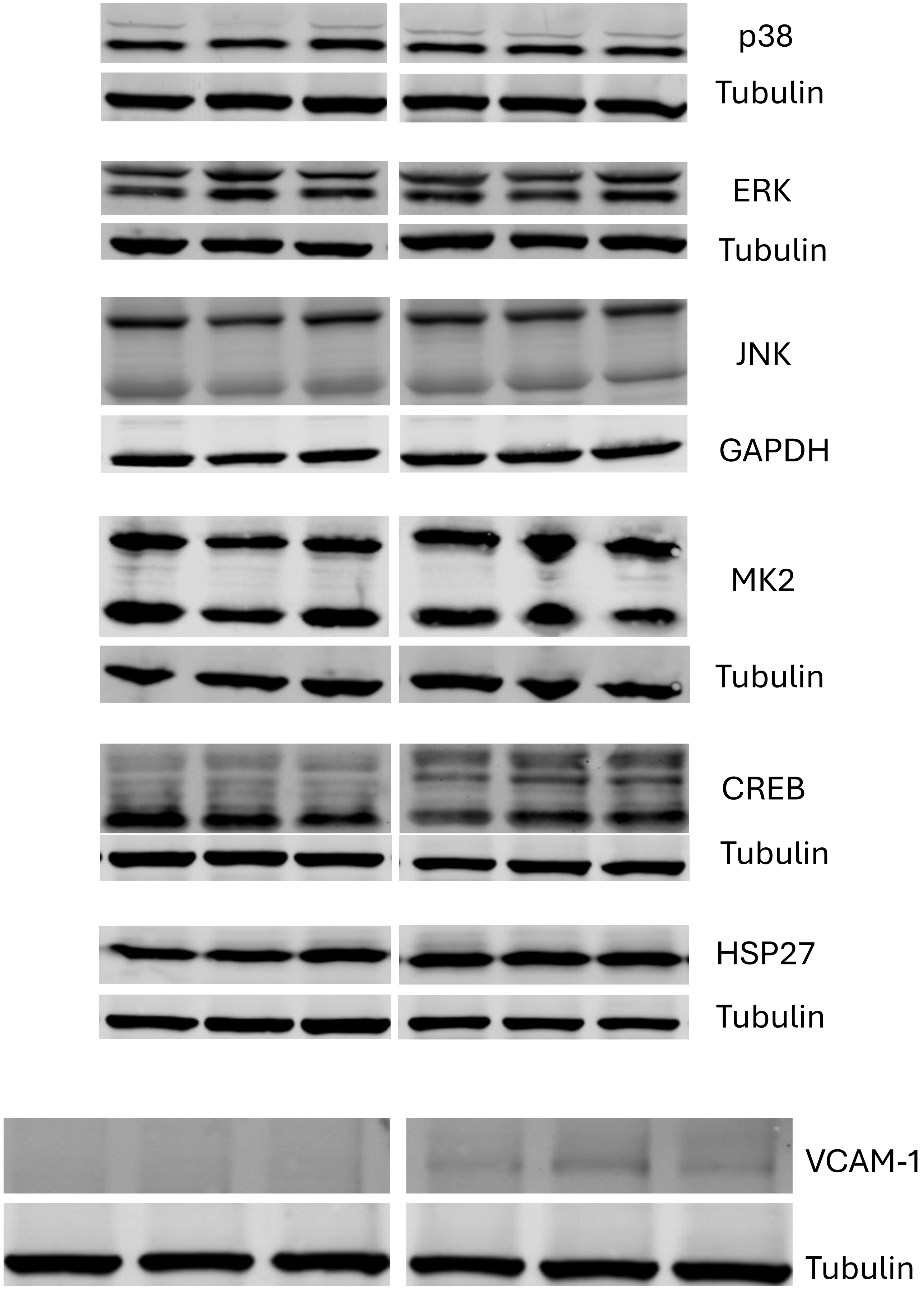

Supplement: Supplementary file 2 — Supplementary material 2 (PDF 2107.3 kb) [file 11033_2026_11504_MOESM2_ESM.pdf]
